# Burden of Stroke in Europe: Thirty-Year Projections of Incidence, Prevalence, Deaths, and Disability-Adjusted Life Years

**DOI:** 10.1161/STROKEAHA.120.029606

**Published:** 2020-07-10

**Authors:** Hatem A. Wafa, Charles D.A. Wolfe, Eva Emmett, Gregory A. Roth, Catherine O. Johnson, Yanzhong Wang

**Affiliations:** 1 School of Population Health and Environmental Sciences, King’s College London, United Kingdom (H.A.W., C.D.A.W., E.E., Y.W.).; 2 National Institute for Health Research (NIHR) Biomedical Research Centre, Guy’s and St Thomas’ NHS Foundation Trust and King’s College London, United Kingdom (H.A.W., C.D.A.W., Y.W.).; 3 National Institute for Health Research (NIHR) Collaboration for Leadership in Applied Health Research and Care (CLAHRC) South London, United Kingdom (H.A.W., C.D.A.W., Y.W.).; 4 Department of Medicine, University of Washington, Seattle (G.A.R.).; 5 Institute of Health Metrics and Evaluation (IHME), University of Washington, Seattle (G.A.R., C.O.J.).

**Keywords:** global burden of disease, health status, incidence, prevalence, stroke

## Abstract

**Background and purpose:**

Prediction of stroke impact provides essential information for healthcare planning and priority setting. We aim to estimate 30-year projections of stroke epidemiology in the European Union using multiple modeling approaches.

**Methods:**

Data on stroke incidence, prevalence, deaths, and disability-adjusted life years in the European Union between 1990 and 2017 were obtained from the global burden of disease study. Their trends over time were modeled using 3 modeling strategies: linear, Poisson, and exponential regressions―adjusted for the gross domestic product per capita, which reflects the impact of economic development on health status. We used the Akaike information criterion for model selection. The 30-year projections up to 2047 were estimated using the best fitting models, with inputs on population projections from the United Nations and gross domestic product per capita prospects from the World Bank. The technique was applied separately by age-sex-country groups for each stroke measure.

**Results:**

In 2017, there were 1.12 million incident strokes in the European Union, 9.53 million stroke survivors, 0.46 million deaths, and 7.06 million disability-adjusted life years lost because of stroke. By 2047, we estimated an additional 40 000 incident strokes (+3%) and 2.58 million prevalent cases (+27%). Conversely, 80 000 fewer deaths (–17%) and 2.31 million fewer disability-adjusted life years lost (–33%) are projected. The largest increase in the age-adjusted incidence and prevalence rates are expected in Lithuania (average annual percentage change, 0.48% and 0.7% respectively), and the greatest reductions in Portugal (–1.57% and –1.3%). Average annual percentage change in mortality rates will range from −2.86% (Estonia) to −0.08% (Lithuania), and disability-adjusted life years’ from −2.77% (Estonia) to −0.23% (Romania).

**Conclusions:**

The number of people living with stroke is estimated to increase by 27% between 2017 and 2047 in the European Union, mainly because of population ageing and improved survival rates. Variations are expected to persist between countries showing opportunities for improvements in prevention and case management particularly in Eastern Europe.

## Abstract 2

Supplemental Digital Content is available in the text.

In the European Union (EU), stroke is the second most common cause of death and a leading cause of adult disability.^1^ It affects ≈1.1 million inhabitants of Europe every year^2^ and causes 440 000 deaths.^3^ In 2017, the cost associated with stroke was estimated at €45 billion, including direct and indirect costs of care provision and productivity loss.^1^ As populations continue to grow and live to an older age, stroke events and their long-term sequelae, and the corresponding costs, are expected to increase dramatically.^4^ Future projection of the burden of stroke is therefore valuable for medium- and long-term planning and organization of stroke services and prevention activities.

Several authors have calculated projections of stroke for a specific region,^5^ country,^6–10^ or internationally,^11–13^ combining demographic projections with estimated future incidence and mortality rates. Those estimates were either based on the most recent age-specific rates^5,9^ or obtained by extrapolation from past trends.^6–8,10–12^ In a 2006 study,^11^ investigators used demographic projections up to 2025 together with WHO estimates of incidence rates to project incidence of stroke in all EU countries, plus Iceland, Norway, and Switzerland.^14^ They forecasted an increase in the number of incident stroke events from 1.1 million in 2000 to over 1.5 million in 2025 if the 2000’s rates remained stable, and a lower but still significant increase to around 1.35 million if rates declined by 2% every 5 years. This approach predicts the number of stroke events that would arise from demographic changes with an arbitrary assumption about epidemiological trends over time. Incorporating significant predictors of stroke risk (eg, hypertension, smoking, and alcohol consumption) into the model can help to reduce uncertainty and improve reliability. However, scarcity of these kinds of data and their future projections across the EU limit the feasibility of such an approach. Incorporating a distal indicator could be an alternative. Previous GBD studies projecting heath outcomes have shown that measures of economic development such as the gross domestic product per capita (GDP) could be a useful proxy for the changes in risk factors and their management, socioeconomic status, and education, among other time-varying indicators.^13,15,16^

We aimed to use GDP as a holistic predictor of stroke events and outcomes to project not only stroke-associated incidence but also prevalence, deaths, and disability-adjusted life years (DALYs) lost over the next 30 years, after accounting for past trends, both overall in the EU and in individual countries. We combined GDP estimates, prepared by the World Bank, with GBD data and the United Nations’ population projections to fit models that would (1) best describe the observed time-trends between 1990 and 2017 and (2) predicts future events up until 2047.

## Methods

All GBD data and materials have been made publicly available at the GBD Network and can be accessed at http://ghdx.healthdata.org/gbd-results-tool.

### Projection Methods

Separate projection models by sex and age groups, in 5-year bands, were developed for 28 European countries to estimate time-trends in 4 stroke measures: incidence, prevalence, deaths, and DALYs lost. Each measure was modeled as a rate using historically observed data between 1990 and 2017. Instead of modeling the effects of multiple covariates, from the limited data that are available, our models predict stroke epidemiological profile as a function of 2 distal variables, which showed clear historical relationship with the outcome measures. These were time in years, a proxy measure which potentially reflects the effect of medical advances, and the average income per capita, measured as GDP and adjusted for purchasing power parity in 2011 US Dollars, a collective measure which indicates the indirect impact of economic development on health status. The former variable (time) captures the effects of accumulating knowledge and medical development, allowing the implementation of more effective health interventions, both preventive and curative, at constant levels of income.^15^ The latter variable (GDP) has shown to be a distant, yet strong, predictor of stroke outcomes.^15,17,18^

Three regression models were performed: linear, exponential, and Poisson. The Akaike information criterion was used to compare the fit of these formulations. In agreement with the Global Burden of Disease (GBD) methodology,^15,16^ model selection suggested that exponential regressions provided the best fit for all measures in almost all age-sex-cohort groups and were therefore used to develop the final models (models parameters are detailed in Tables I and II in the Data Supplement).

The final regression equations took the following form:


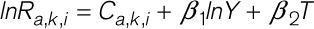


Where 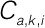 is a constant term; 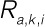 is the outcome rate for age group *a*, sex *k*, and country *i*; and *Y* and *T* denote GDP per person and time in years, respectively. Those age-sex-country-specific models used 2018 to 2047 GDP inputs from the World Bank to generate 30-year forecasts of rates beyond the reference year (2017). The methods used are generally similar to previous GBD studies,^13,15,16^ with more systematic procedure for model selection in addition to using updated inputs of stroke-specific data not only for incidence and mortality but also prevalence and DALYs.

Our forecast projected the absolute numbers of stroke incidence, prevalence, deaths, and DALYs by multiplying the projected rates in 2018 to 2047 by the corresponding age-, sex-, year-, and country-specific population projections. Estimates were then aggregated for presentation of the results in individual countries and overall in the EU. In addition, we calculated the absolute number of events that would occur if rates remained stable (baseline), decreased annually by 1% (optimistic), and increased annually by 1% (pessimistic) with reference to the 2017 observed rates. We reported age-adjusted incidence and mortality rates per 100 000 person-years, and prevalence and DALYs per 10 000 people using the direct method of standardization and the updated European Standard Population (2013) as a reference.^19^ Finally, the average annual percentage changes (AAPC) in the crude and age-adjusted rates were calculated by dividing the relative change between 2017 and 2047 by the number of years. All analyses were performed using the statistical software R version 3.5.0.

### Model Validation and Forecasting Analysis

We ran internal model validation and forecasting analysis comparing our methods to the most widely used demographic approach.^20^ We fitted our models using only data from 1990 to 2007 to forecast stroke outcomes during 2008 to 2017. Out-of-sample validation forecasts for 2008 to 2017 were then compared with the observed data in the same period. Accuracy was assessed over the test period using the root-mean-squared error.

Despite the scarcity of risk factors data, additional models were fitted in 2 countries (Sweden and the United Kingdom) where more complete data could be obtained from GBD during the study period. In these risk factors models, the changing prevalence of diabetes mellitus, hypertension, and atrial fibrillation were incorporated as direct predictors of stroke outcomes. Again, models were fitted to 1990 to 2007 data, then information on demography and risk factors during 2008 to 2017 was used to guide the forecasts during that period. Results were then compared to our models’ using GDP as a distant predictor of stroke outcomes (Figures I and II in Data Supplement).

### Data Sources

We used the results of GBD 2017 to estimate patterns of stroke incidence, prevalence, deaths, and DALYs between 1990 and 2017.^21^ Count data were obtained on a national scale for 28 EU countries by age and sex groups. A wide range of data sources was used to estimate these measures which are described in detail previously.^22–25^ In brief, for the selected countries, systematic review of the literature was performed to identify ideal population-based studies. Only studies that used the WHO’s definition of stroke^26^ with complete case ascertainment were included. Death registration data were provided to the WHO by Member States. In addition, population-based epidemiological studies, disease registers, and surveillance systems contributed to estimation of stroke mortality.^13^ Estimation of DALYs was based on calculation of 2 components: years of life lost because of death, and years lived with disability. Detailed methodology is described elsewhere.^27,28^ One DALY represents a year of healthy life lost. All GBD data sources can be accessed via the GBD 2017 Data Input Sources Tool.

Population estimates and projections for each country were obtained from the United Nations (UN) Department of Economic and Social Affairs/Population Division.^29^ Future prospects were made according to a framework which accounts for the 3 demographic components of change―fertility, mortality, and international migration.^30^ We used the medium-variant assumption in our analyses, which assumes a decline in the fertility for countries where large families are still prevalent and a continuous decline in death rates throughout the age range. Historical and future indicators of economic development, as measured in GDP per capita, were obtained from the World Bank and the OECD online toolkits.^31,32^ All data are deidentified and publicly available, and hence no ethics approval was necessary. Interactive online tools are also available to explore all data sources in detail.

GBD data on stroke incidence, prevalence, deaths, and DALYs were complete for all countries across the years (1990–2017). GDP data from the World Bank were missing for a few countries in the early years: 1990 to 1991 for Slovakia; 1990 to 1994 for Croatia, Estonia, Latvia, Lithuania, and Slovenia; and 1990 to 2000 for Malta. Autoregressive integrated moving average models were fitted to interpolate these missing values.

## Results

**Figure 1.**
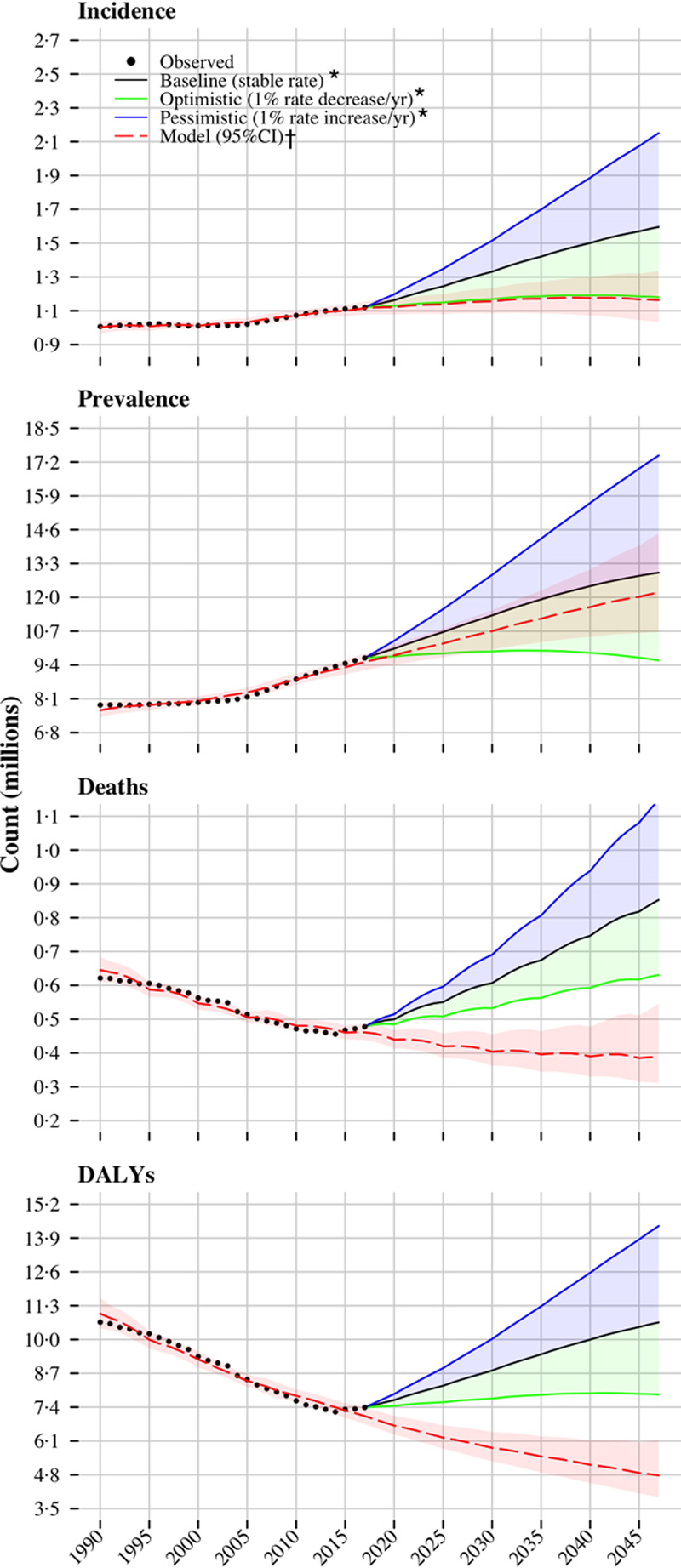
**Historical trends and future trajectories of stroke incidence, prevalence, deaths, and disability-adjusted life years (DALYs) in the European Union since 1990 and up to 2047.** *With reference to 2017 observed rates. †Constructed as detailed in Methods section.

In 2017, ≈509 million people were residents in the EU. There were an estimated 1.12 million cases of incident stroke, 9.53 million prevalent stroke cases, 0.46 million stroke deaths, and 7.06 million DALYs lost (Table). By 2047, the population size is expected to remain relatively stable; however, there will be an additional 40 000 incident stroke cases (3% increase) and 2.58 million prevalent cases (27% increase). This is largely because of the projected changes in the population age structure, and, in particular, by an increase in the number of residents aged ≥70 years old (comprising 23% of the population in 2047 compared with 14% in 2017), in which stroke risk is the highest. Conversely, the declining trends in deaths and DALYs between 1990 and 2017 are expected to continue through 2047 (Figure 1), resulting in ≈80 000 fewer deaths (17% reduction) and 2.31 million fewer DALYs (33% reduction). The age-adjusted rates on the other hand for the 4 epidemiological measures are predicted to decline: 26% for incidence, 9% for prevalence, and 55% for both mortality and DALYs lost.

**Table.**
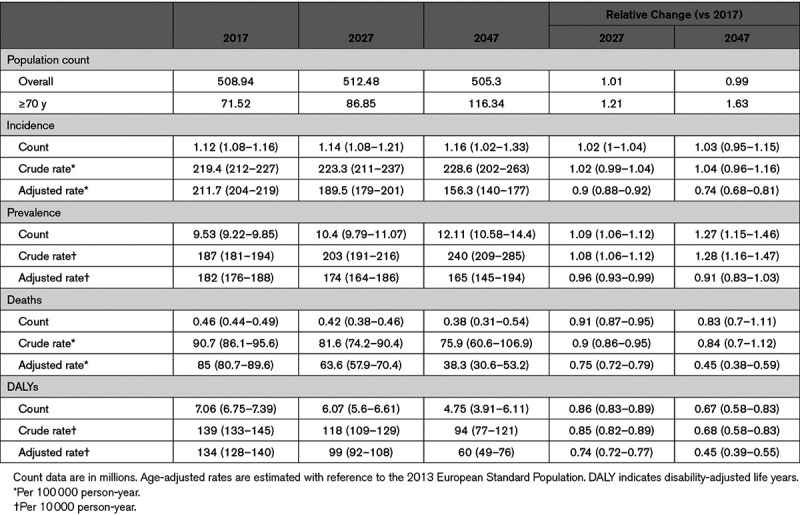
Changes in Population Count and Stroke Incidence, Prevalence, Deaths, and DALYs in the EU Between 2017 and 2047

**Figure 2.**
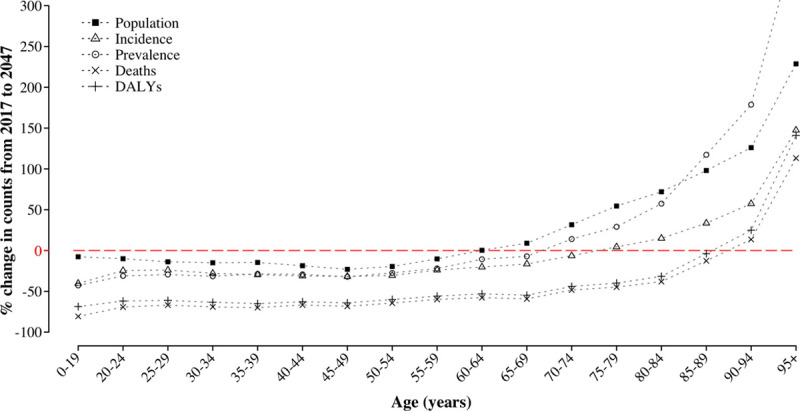
**Projected change in population, incidence, prevalence, deaths, and disability-adjusted life years disability-adjusted life years (DALYs) count by age group in the European Union (2047 vs 2017).**

Figure 2 illustrates the percentage changes between 2017 and 2047 in the counts of the EU population and stroke incidence, prevalence, deaths, and DALYs by age cohorts. Greater magnitude of change for older age groups is demonstrated for all measures, which reflects population aging and a larger, rising burden of stroke in older people. The expected increase in incident and prevalent stroke cases will be exclusive to those aged ≥70 years, whereas a reduction will be observed in the younger age groups. On the other hand, mortality and DALYs lost because of stroke will decline in all ages except people ≥90 years old.

A breakdown of the absolute numbers by countries are provided in Figures III through VI in Data Supplement. While several countries will experience a varying increase in numbers of incident strokes, others will have either no change or a decrease in numbers (Figure III in the Data Supplement). All but 7 countries (Estonia, Portugal, Italy, Hungary, Latvia, Romania, and Czech Republic) will experience an increase in the number of stroke survivors, and almost all countries will see a decline in the number of deaths and DALYs lost.

**Figure 3.**
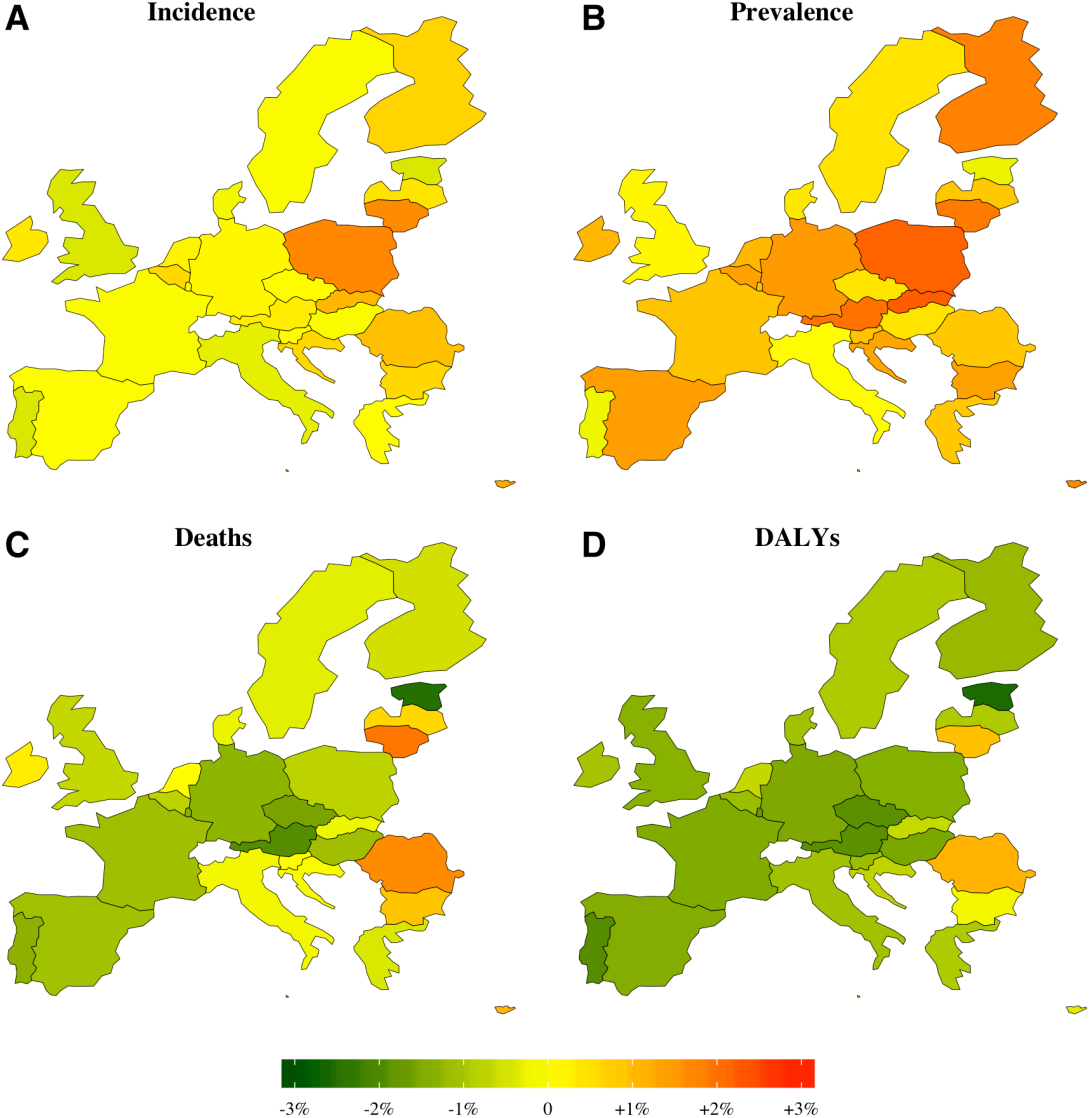
**Projected average annual percentage change in the crude rates of incidence, prevalence, death, and disability-adjusted life years (DALYs) during 2018 to 2047.**

**Figure 4.**
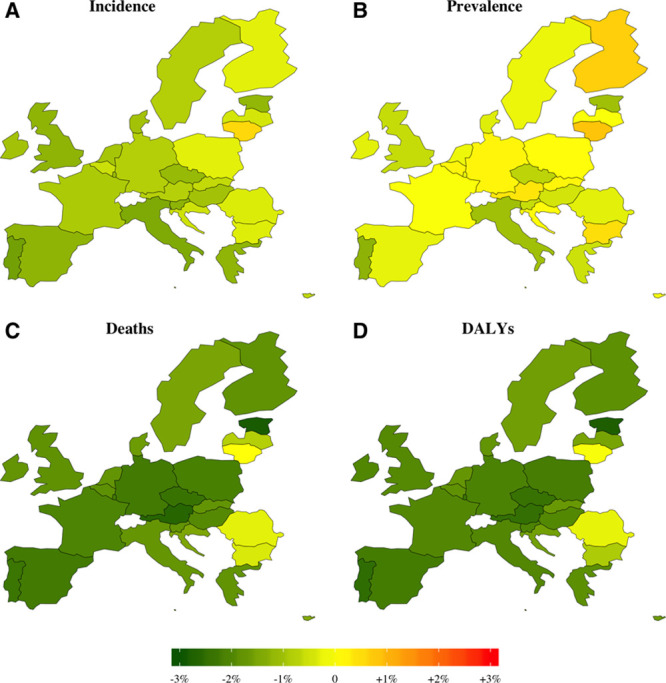
**Projected average annual percentage change in the age-standardized rates of incidence, prevalence, death, and disability-adjusted life years (DALYs) during 2018 to 2047.**

Projected crude and age-adjusted rates give account of future stroke epidemiology regardless of variations in population size and/or age structure. Figure 3 demonstrates the estimated AAPC in the crude rates of stroke between 2018 and 2047. Most countries will continue past trends of increasing crude incidence and prevalence rates, while mortality and DALYs lost will decrease. Nevertheless, the age-adjusted rates are estimated to decline over the next decades in almost all countries, and an East-West gradient seems to persist (Figure 4). In 2047, our models predict age-adjusted rates ranging from 467 (Lithuania) to 92/100 000 (Italy) for incidence, from 428 (Lithuania) to 73/10 000 (Italy) for prevalence, from 267 (Bulgaria) to 10/100 000 (Austria) for deaths, and from 429 (Romania) to 23/10 000 (Austria) for DALYs lost (Figures VII through X in the Data Supplement). Between 2018 and 2047, the age-adjusted AAPC will range, for incidence, from –1.57 % (Portugal) to 0.48% (Lithuania); prevalence, –1.3 (Portugal) to 0.7% (Lithuania); deaths, –2.86% (Estonia) to 0.08% (Lithuania); and DALYs, –2.77% (Estonia) to –0.23% (Romania).

**Figure 5.**
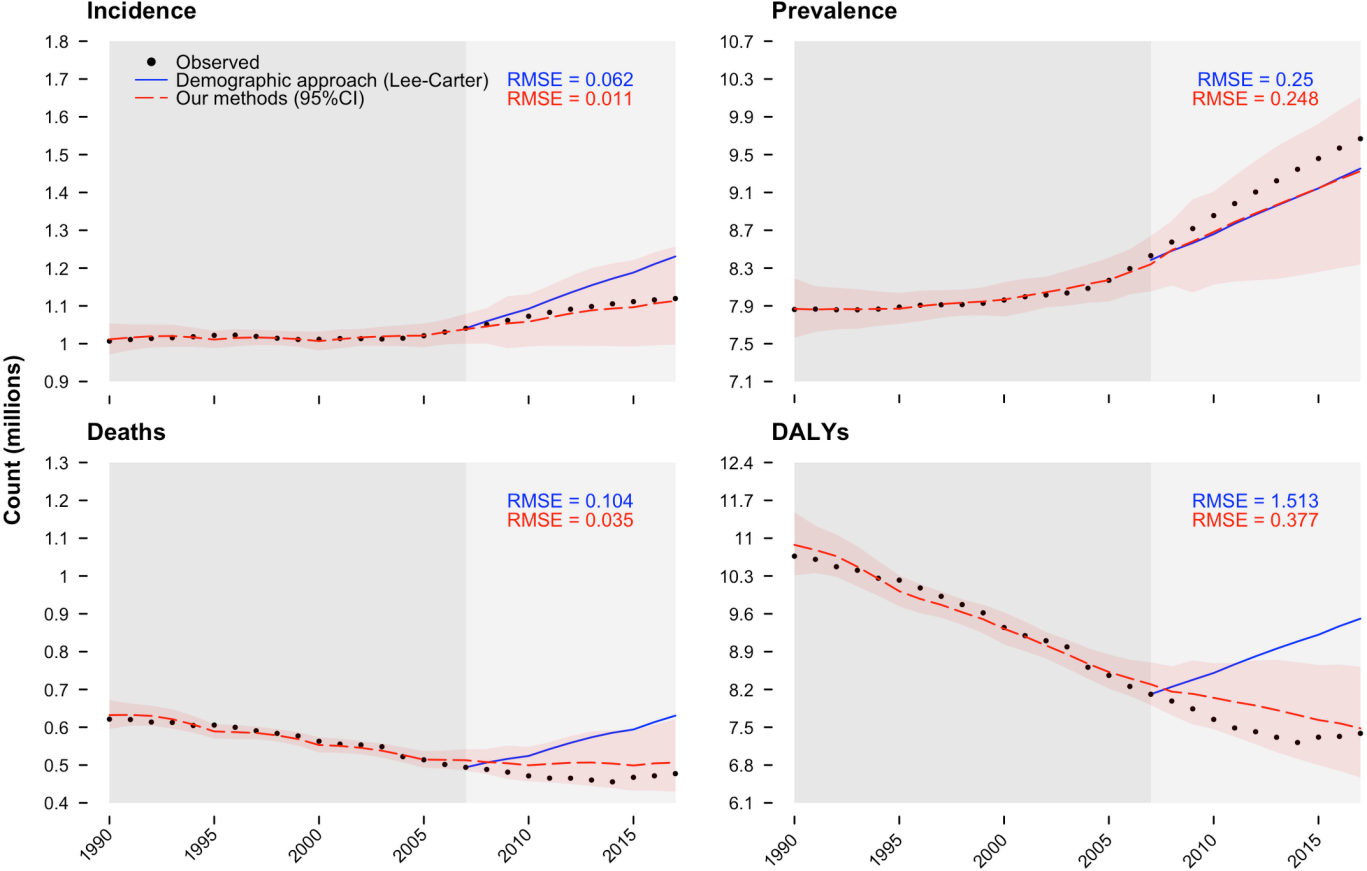
**Prediction performance of different models in the European Union during a test period (2008–2017).** RMSE indicates root mean squared error which reflects the average deviation of the predictions from the observed values over evaluation years (2008–2017).

Comparison of out-of-sample prediction and past data are shown in Figure 5. Results show that our models had lower prediction errors than Lee-Carther approach during the test period, indicating superior performance. For instance, our methods predicted stroke incidence during 2008 to 2017 with a mean error of 0.011 million cases whereas Lee-Carter mean error was 0.062 million strokes. Furthermore, our forecasts during this test period were similar to those obtained from risk factors models in the United Kingdom and Belgium (Figures I and II, respectively in the Data Supplement).

## Discussion

This study provides country-specific projections of stroke burden in the EU up to 2047 using multiple modeling techniques building on GBD estimates. Unlike conventional projection approaches, not only did the current analysis account for demographic and epidemiological changes, but also prospects of economic development (measured in GDP per capita), a proxy measure for the complex changes in lifestyle, risk factors, and their management, and other environmental and behavioral factors.^15,17,18^ Previous GBD investigators carried out projections of mortality and DALYs for disease-specific^13,33^ and compiled clusters of cause-of-death,^15,16^ and presented the results on regional and global levels.^13,15,16^ In the current study, we analyzed 4 epidemiological measures for stroke in 28 EU countries, applying more rigourous model selection methodology, and using detailed and updated information to guide projections and produce more reliable estimates. Between 2017 and 2047, we predict an increase in the absolute count of stroke events in the EU by 3% (from 1.12 to 1.16 million), and stroke survivors by 27% (9.53 to 12.11 million), despite a projected decline in the corresponding age-standardized rates. Deaths and DALYs because of stroke are expected to decrease by 17% (0.46–0.38 million) and 33% (7.06–4.75 million), respectively.

Previous studies conducted in Europe^5–10,34^ and other high-income countries^35–37^ also predicted rises in stroke numbers. The Stroke Alliance for Europe anticipated a 34% increase in incident stroke events between 2015 and 2035.^34^ In contrast, the current study forecasts a modest increase by 3% between 2017 and 2047. Methodological differences is likely to explain such disparity. Stroke Alliance for Europe and other previous projection studies were either based on a stable rates assumption or linear extrapolation of past trends. The former is likely to overestimate stroke events whereas the latter could lead to implausible increase/decrease in rates and the subsequent counts, and therefore these approaches are suboptimal. Furthermore, Stroke Alliance for Europe used the Eurostat population data which predict significant population ageing and growth, whereas the UN data, used in the current study, project a remarkable aging but a relatively stable population size. On the other hand, the age-adjusted incidence rates produced by our models are not divergent from past trends, an AAPC of –0.87% between 2018 and 2047 versus –1% reported in developed countries during 1970 to 2008.^38^ Truelsen et al^11^ used an arbitrary change of ±2%/5 years to project a range of stroke incidence in the EU between 2000 and 2025. The results, however, appeared to overestimate stroke events―the projected figures are larger than those already observed during 2000 to 2017. For instance, in 2015, ≈1.28 million new strokes were projected even under the optimistic scenario (−2%/5 years), which is still larger than the 1.1 million cases reported by GBD for the same year.^21^

By 2047, 2.4% of the EU population are estimated to have had a stroke―an increase of 28% compared with 2017 which may be explained as follows. As demographics change over time, older people will constitute a larger proportion of the EU population despite the relatively stable overall count projected for the future. Eventually, this factor will contribute greater number of patients with stroke who will have a greater chance of surviving the initial event for 2 reasons. First, many of these strokes will be milder in nature because of the ongoing advances and widespread implementation of primary prevention strategies,^39,40^ and second, patients in the future are more likely to receive better care and treatment both in the acute and long-term stages after stroke. Consequently, a shift in stroke burden from mortality to morbidity is likely to be observed in the future. If disability levels among stroke survivors remain unchanged over the next decades, then the demand for rehabilitation and long-term care will increase by 27% in the EU. Economically, this increased demand will have to be borne by a smaller working age population (currently defined as those aged 15–64),^41^ which will in turn have implications in terms of increased workload and per capita financial burden. While several studies predict rising future costs of stroke,^8,9^ there is some evidence that certain treatments (eg, thrombolysis, early supported discharge) can contribute to containing these costs.^8,42^

For most EU countries, significant declines in stroke mortality have been reported since 1970.^43–45^ One study calculated an AAPC of −2.7% in the age-adjusted mortality rate between 1980 and 2016 across Europe,^45^ and a range of −1% and −4% was reported in seven Western European countries between 1980 and 2005.^12^ These estimates are comparable to our historical AAPC of –3.3% observed during 1990 to 2017, and our projected figure of −1.8% within the next 30 years (2018–2047). Indeed, past improvements in mortality are linked to better survival rates, partly because of advances in therapeutic options and acute management (eg, thrombolysis treatment and stroke unit care).^46,47^ Wider application of these approaches together with implementation of newer treatment methods (eg, thrombectomy and centralized care provision)^48–50^ might therefore bring further reductions. However, mortality is not only determined by survival and case-fatality but also by incidence rate. Although it is difficult to ascertain which measure has a greater influence on mortality in absence of reliable survival data, the decline in stroke incidence and mortality suggests a greater contribution of incidence on mortality reduction.^13,38^

Variation across EU countries in stroke incidence, prevalence, deaths, and DALYs has been documented in the past^2,43,45,51^ and is noted in the current study. An East-West gradient will be observed in the future, and the most striking increase in the age-adjusted rate of stroke prevalence is expected in Lithuania (AAPC, 0.7%). It is also the only EU country which is predicted to witness an increase in stroke incidence (0.48%). Portugal on the other hand is estimated to have the greatest reductions in incidence (−1.57%) and prevalence (−1.3%). Possible improvement in health systems, case detection, and clinical diagnosis might have contributed to the apparent changes in stroke incidence and subsequent prevalence rates. However, differences in exposure to cardiovascular risk factors over time and their level of control could also account for the observed and projected geographic variations. For instance, Portugal achieved one of the greatest reductions in smoking rates between 1990 and 2014, whereas Lithuania is the only EU country which had a remarkable increase in alcohol consumption rates during the same period.^52^ In Estonia, significant reductions in stroke mortality and DALYs were observed (1990–2017) and are estimated to extrapolate into the future (AAPC, −2.86% and −2.77%, respectively). It is also one of the countries where prescription of antihypertensive and lipid-modifying drugs soared between 2000 and 2013,^52^ which might reflect improved primary and secondary prevention and hence reduced incidence and case-fatality. While some countries (eg, Portugal and Greece) had relatively higher rates of stroke incidence in the past and therefore larger capacity for improvement and steeper declines (1990–2017), others, such as Italy, France, and the United Kingdom, achieved further reductions from already lower rates. On the other hand, most Eastern European countries (eg, Lithuania and Romania), despite having the highest rates in the past, achieved modest declines, or even increases, which might indicate less efficient interventions. Such historical patterns will reflect in the future and are expected to persist. Our projections give an indication of the rates that could potentially be achieved across the EU with sustained public health efforts and suggest that further improvement is possible in most countries.

The current analysis combines large, international, and standardizsed datasets that are highly comparable; however, we emphasise that our estimates are based on a set of assumptions about future demographic, epidemiological, and economic trends. Therefore, our projections are simply visioning what the future may unfold if these assumptions hold true, and we emphasize that they need to be interpreted with this in mind. We did not take explicit account of trends in major stroke risk factors (eg, hypertension, smoking, and poor diet) because of scarcity of the data. If exposure to stroke risk factors increase, rather than decrease, with economic development, then our projections will be underestimates. More comprehensive projection models that specifically take account of these risk factors with further stratification by ethnic groups might be valuable. A recent GBD study developed a novel forcasting model which incorporates the relationships between the independent drivers of health captured within GBD.^33^ The model was used to estimate all-cause and disease-specific mortality, years of life lost, and life expectancy up until 2040, and the results were presented on regional and global scales. Adopting such an approach could be useful to better forecast other important disease-specific measures like incidence, prevalence, and DALYs by incorporating relevant and vital inputs to improve projections accuracy. We finally acknowledge that data quality may vary across the EU, which could make some estimates less certain than others particularly in Eastern European countries where epidemiological studies are less frequent.^38^ Nevertheless, this study provides a useful perspective on population health trends, which could have implications on health policy and priority setting.

In conclusion, the absolute burden of stroke was increasing and is expected to continue to increase over the next 30 years in most EU countries, particularly in Eastern states. With an estimated 27% increase in the number of people surviving a stroke in Europe, in combination with a reduced proportion of people at working ages, there is an imperative to make greater efforts to prevent stroke. This would be the most effective strategy to reduce the anticipated financial and logistic challenges facing countries with an already stressed healthcare systems.

## Sources of Funding

The National Institute for Health Research (NIHR) Collaboration for Leadership in Applied Health Research and Care South London at King’s College Hospital National Health Services (NHS) Foundation Trust, the NIHR Biomedical Research Centre based at Guy’s and St Thomas’ NHS Foundation Trust and King’s College London, and the European Union’s Horizon 2020 Research and Innovation Programme under grant agreement No. 754517. Dr Johnson received funding from Bill and Melinda Gates Foundation.

## Disclosures

None.

## Supplementary Material



## References

[1] Wilkins E Wilson L Wickramasinghe K Bhatnagar P Leal J Luengo-Fernandez R Burns R Rayner M Townsend N European Cardiovascular Disease Statistics 2017 2017 Brussels European Heart Network

[2] Béjot Y Bailly H Durier J Giroud M Epidemiology of stroke in Europe and trends for the 21^st^ century. Presse Med 2016 45 12 pt 2 e391–e398 doi: 10.1016/j.lpm.2016.10.003 27816343 10.1016/j.lpm.2016.10.003

[3] OECD (2016). Mortality from heart disease and stroke.. In: Health at a Glance: Europe 2016: State of Health in the EU Cycle.

[4] Bennett DA Krishnamurthi RV Barker-Collo S Forouzanfar MH Naghavi M Connor M Lawes CM Moran AE Anderson LM Roth GA; Global Burden of Diseases, Injuries, and Risk Factors 2010 Study Stroke Expert Group The global burden of ischemic stroke: findings of the GBD 2010 study. Glob Heart 2014 9 107–112 doi: 10.1016/j.gheart.2014.01.001 25432120 10.1016/j.gheart.2014.01.001

[5] Foerch C Misselwitz B Sitzer M Steinmetz H Neumann-Haefelin T; Hesse Stroke Study Group The projected burden of stroke in the German federal state of Hesse up to the year 2050. Dtsch Arztebl Int 2008 105 467–473 doi: 10.3238/arztebl.2008.0467 19626195 10.3238/arztebl.2008.0467 PMC2696913

[6] Struijs JN van Genugten ML Evers SM Ament AJ Baan CA van den Bos GA Modeling the future burden of stroke in The Netherlands: impact of aging, smoking, and hypertension. Stroke 2005 36 1648–1655 doi: 10.1161/01.STR.0000173221.37568.d2 16002757 10.1161/01.STR.0000173221.37568.d2

[7] Sivenius J Torppa J Tuomilehto J Immonen-Räihä P Kaarisalo M Sarti C Kuulasmaa K Mähönen M Lehtonen A Salomaa V Modelling the burden of stroke in Finland until 2030. Int J Stroke 2009 4 340–345 doi: 10.1111/j.1747-4949.2009.00330.x 19765121 10.1111/j.1747-4949.2009.00330.x

[8] Smith S Horgan F Sexton E Cowman S Hickey A Kelly P McGee H Murphy S O’Neill D Royston M The future cost of stroke in Ireland: an analysis of the potential impact of demographic change and implementation of evidence-based therapies. Age Ageing 2013 42 299–306 doi: 10.1093/ageing/afs192 23302602 10.1093/ageing/afs192

[9] Kolominsky-Rabas PL Heuschmann PU Marschall D Emmert M Baltzer N Neundörfer B Schöffski O Krobot KJ Lifetime cost of ischemic stroke in Germany: results and national projections from a population-based stroke registry: the Erlangen Stroke Project. Stroke 2006 37 1179–1183 doi: 10.1161/01.STR.0000217450.21310.90 16574918 10.1161/01.STR.0000217450.21310.90

[10] Hallström B Jönsson AC Nerbrand C Norrving B Lindgren A Stroke incidence and survival in the beginning of the 21^st^ century in southern Sweden: comparisons with the late 20^th^ century and projections into the future. Stroke 2008 39 10–15 doi: 10.1161/STROKEAHA.107.491779 18063825 10.1161/STROKEAHA.107.491779

[11] Truelsen T Piechowski-Jóźwiak B Bonita R Mathers C Bogousslavsky J Boysen G Stroke incidence and prevalence in Europe: a review of available data. Eur J Neurol 2006 13 581–598 doi: 10.1111/j.1468-1331.2006.01138.x 16796582 10.1111/j.1468-1331.2006.01138.x

[12] Kunst AE Amiri M Janssen F The decline in stroke mortality: exploration of future trends in 7 Western European countries. Stroke 2011 42 2126–2130 doi: 10.1161/STROKEAHA.110.599712 21700943 10.1161/STROKEAHA.110.599712

[13] Strong K Mathers C Bonita R Preventing stroke: saving lives around the world. Lancet Neurol 2007 6 182–187 doi: 10.1016/S1474-4422(07)70031-5 17239805 10.1016/S1474-4422(07)70031-5

[14] Truelsen T Begg S Mathers CD Satoh T Global burden of cerebrovascular disease in the year 2000. 2002 In: GBD 2000 Working Paper Geneva, Switzerland WHO

[15] Mathers CD Loncar D Projections of global mortality and burden of disease from 2002 to 2030. PLoS Med 2006 3 e442 doi: 10.1371/journal.pmed.0030442 17132052 10.1371/journal.pmed.0030442 PMC1664601

[16] Murray CJ Lopez AD Alternative projections of mortality and disability by cause 1990-2020: Global Burden of Disease Study. Lancet 1997 349 1498–1504 doi: 10.1016/S0140-6736(96)07492-2 9167458 10.1016/S0140-6736(96)07492-2

[17] Asplund K What MONICA told us about stroke. Lancet Neurol 2005 4 64–68 doi: 10.1016/S1474-4422(04)00967-6 15620858 10.1016/S1474-4422(04)00967-6

[18] Zahra A Lee EW Sun LY Park JH Cardiovascular disease and diabetes mortality, and their relation to socio-economical, environmental, and health behavioural factors in worldwide view. Public Health 2015 129 385–395 doi: 10.1016/j.puhe.2015.01.013 25724438 10.1016/j.puhe.2015.01.013

[19] Ahmad OB Boschi-Pinto C Lopez AD Murray CJ Lozano R Inoue M Age Standardization of Rates: a New WHO Standard 2001 9 Geneva World Health Organization 10

[20] Lee RD Carter LR Modeling and forecasting U.S. mortality. J Am Stat Assoc 1992 87 659–671

[21] Global Burden of Disease Collaborative Network (2017). Global Burden of Disease Study 2017 (GBD 2017) Results.

[22] Feigin VL Forouzanfar MH Krishnamurthi R Mensah GA Connor M Bennett DA Moran AE Sacco RL Anderson L Truelsen T; Global Burden of Diseases, Injuries, and Risk Factors Study 2010 (GBD 2010) and the GBD Stroke Experts Group Global and regional burden of stroke during 1990-2010: findings from the Global Burden of Disease Study 2010. Lancet 2014 383 245–254 doi: 10.1016/s0140-6736(13)61953-4 24449944 10.1016/s0140-6736(13)61953-4 PMC4181600

[23] James SL Abate D Abate KH Abay SM Abbafati C Abbasi N Abbastabar H Abd-Allah F Abdela J Abdelalim A Abdollahpour I Global, regional, and national incidence, prevalence, and years lived with disability for 354 diseases and injuries for 195 countries and territories, 1990-2017: a systematic analysis for the Global Burden of Disease Study 2017. Lancet 2018 392 1789–1858 doi: 10.1016/S0140-6736(18)32279-7 30496104 10.1016/S0140-6736(18)32279-7 PMC6227754

[24] Johnson CO Nguyen M Roth GA Nichols E Alam T Abate D Global, regional, and national burden of stroke, 1990-2016: a systematic analysis for the Global Burden of Disease Study 2016. Lancet Neurol 2019 18 439–458 30871944 10.1016/S1474-4422(19)30034-1 PMC6494974

[25] Naghavi M Abajobir AA Abbafati C Abbas KM Abd-Allah F Abera SF Global, regional, and national age-sex specific mortality for 264 causes of death, 1980-2016: a systematic analysis for the Global Burden of Disease Study 2016. Lancet 2017 390 1151–1210 doi: 10.1016/S0140-6736(17)32152-9 28919116 10.1016/S0140-6736(17)32152-9 PMC5605883

[26] Hatano S Experience from a multicentre stroke register: a preliminary report. Bull World Health Organ 1976 54 541–553 1088404 PMC2366492

[27] Murray CJ Ezzati M Flaxman AD Lim S Lozano R Michaud C Naghavi M Salomon JA Shibuya K Vos T GBD 2010: design, definitions, and metrics. Lancet 2012 380 2063–2066 doi: 10.1016/S0140-6736(12)61899-6 23245602 10.1016/S0140-6736(12)61899-6

[28] Murray CJ Vos T Lozano R Naghavi M Flaxman AD Michaud C Ezzati M Shibuya K Salomon JA Abdalla S Disability-adjusted life years (DALYs) for 291 diseases and injuries in 21 regions, 1990-2010: a systematic analysis for the Global Burden of Disease Study 2010. Lancet 2012 380 2197–2223 doi: 10.1016/S0140-6736(12)61689-4 23245608 10.1016/S0140-6736(12)61689-4

[29] United Nations, Department of Economic and Social Affairs, Population Division (2017). World Population Prospects: The 2017 Revision, DVD Edition.

[30] United Nations, Department of Economic and Social Affairs, Population Division (2017). World Population Prospects: The 2017 Revision, Methodology of the United Nations Population Estimates and Projections, Working Paper No. ESA/P/WP.250.

[31] OECD (2014). Long-term baseline projections, No. 95 (edition 2014).

[32] The World Bank (2019). World Development Indicators (WDI).. http://databank.worldbank.org/data/reports.aspx?source=2&series=NY.GDP.PCAP.PP.KD&country=#.

[33] Foreman KJ Marquez N Dolgert A Fukutaki K Fullman N McGaughey M Pletcher MA Smith AE Tang K Yuan CW Forecasting life expectancy, years of life lost, and all-cause and cause-specific mortality for 250 causes of death: reference and alternative scenarios for 2016-40 for 195 countries and territories. Lancet 2018 392 2052–2090 doi: 10.1016/S0140-6736(18)31694-5 30340847 10.1016/S0140-6736(18)31694-5 PMC6227505

[34] Stroke Alliance for Europe (SAFE) (2017). The burden of stroke in Europe: The challenge for policy makers.. https://www.stroke.org.uk/sites/default/files/the_burden_of_stroke_in_europe_-_challenges_for_policy_makers.pdf.

[35] Feigin VL Krishnamurthi RV Barker-Collo S McPherson KM Barber PA Parag V Arroll B Bennett DA Tobias M Jones A; ARCOS IV Group 30-year trends in stroke rates and outcome in Auckland, New Zealand (1981-2012): a multi-ethnic population-based series of studies. PLoS One 2015 10 e0134609 doi: 10.1371/journal.pone.0134609 26291829 10.1371/journal.pone.0134609 PMC4546383

[36] Pearson-Stuttard J Guzman-Castillo M Penalvo JL Rehm CD Afshin A Danaei G Kypridemos C Gaziano T Mozaffarian D Capewell S Modeling future cardiovascular disease mortality in the United States: national trends and racial and ethnic disparities. Circulation 2016 133 967–978 doi: 10.1161/CIRCULATIONAHA.115.019904 26846769 10.1161/CIRCULATIONAHA.115.019904 PMC4783256

[37] Tobias M Cheung J Carter K Anderson C Feigin VL Stroke surveillance: population-based estimates and projections for New Zealand. Aust N Z J Public Health 2007 31 520–525 doi: 10.1111/j.1753-6405.2007.00136.x 18081570 10.1111/j.1753-6405.2007.00136.x

[38] Feigin VL Lawes CM Bennett DA Barker-Collo SL Parag V Worldwide stroke incidence and early case fatality reported in 56 population-based studies: a systematic review. Lancet Neurol 2009 8 355–369 doi: 10.1016/S1474-4422(09)70025-0 19233729 10.1016/S1474-4422(09)70025-0

[39] Rothwell PM Coull AJ Giles MF Howard SC Silver LE Bull LM Gutnikov SA Edwards P Mant D Sackley CM; Oxford Vascular Study Change in stroke incidence, mortality, case-fatality, severity, and risk factors in Oxfordshire, UK from 1981 to 2004 (Oxford Vascular Study). Lancet 2004 363 1925–1933 doi: 10.1016/S0140-6736(04)16405-2 15194251 10.1016/S0140-6736(04)16405-2

[40] Wafa HA Wolfe CDA Bhalla A Wang Y Long-term trends in death and dependence after ischaemic strokes: a retrospective cohort study using the South London Stroke Register (SLSR). PLoS Med 2020 17 e1003048 doi: 10.1371/journal.pmed.1003048 32163411 10.1371/journal.pmed.1003048 PMC7067375

[41] OECD (2019). Working age population (indicator). https://data.oecd.org/pop/working-age-population.htm.

[42] Farzadfar F Finucane MM Danaei G Pelizzari PM Cowan MJ Paciorek CJ Singh GM Lin JK Stevens GA Riley LM; Global Burden of Metabolic Risk Factors of Chronic Diseases Collaborating Group (Cholesterol) National, regional, and global trends in serum total cholesterol since 1980: systematic analysis of health examination surveys and epidemiological studies with 321 country-years and 3·0 million participants. Lancet 2011 377 578–586 doi: 10.1016/S0140-6736(10)62038-7 21295847 10.1016/S0140-6736(10)62038-7

[43] Helis E Augustincic L Steiner S Chen L Turton P Fodor JG Time trends in cardiovascular and all-cause mortality in the ‘old’ and ‘new’ European Union countries. Eur J Cardiovasc Prev Rehabil 2011 18 347–359 doi: 10.1177/1741826710389361 21450659 10.1177/1741826710389361

[44] Sarti C Stegmayr B Tolonen H Mähönen M Tuomilehto J Asplund K; WHO MONICA Project Are changes in mortality from stroke caused by changes in stroke event rates or case fatality? Results from the WHO MONICA Project. Stroke 2003 34 1833–1840 doi: 10.1161/01.STR.0000081224.15480.52 12855832 10.1161/01.STR.0000081224.15480.52

[45] Shah R Wilkins E Nichols M Kelly P El-Sadi F Wright FL Townsend N Epidemiology report: trends in sex-specific cerebrovascular disease mortality in Europe based on WHO mortality data. Eur Heart J 2019 40 755–764 doi: 10.1093/eurheartj/ehy378 30124820 10.1093/eurheartj/ehy378 PMC6396027

[46] Wardlaw JM Murray V Berge E del Zoppo G Sandercock P Lindley RL Cohen G Recombinant tissue plasminogen activator for acute ischaemic stroke: an updated systematic review and meta-analysis. Lancet 2012 379 2364–2372 doi: 10.1016/S0140-6736(12)60738-7 22632907 10.1016/S0140-6736(12)60738-7 PMC3386494

[47] Berge E Cohen G Roaldsen MB Lundström E Isaksson E Rudberg AS Slot KB Forbes J Smith J Drever J; IST-3 Collaborative Group Effects of alteplase on survival after ischaemic stroke (IST-3): 3 year follow-up of a randomised, controlled, open-label trial. Lancet Neurol 2016 15 1028–1034 doi: 10.1016/S1474-4422(16)30139-9 27450474 10.1016/S1474-4422(16)30139-9

[48] Campbell BCV Mitchell PJ Churilov L Keshtkaran M Hong KS Kleinig TJ Dewey HM Yassi N Yan B Dowling RJ; EXTEND-IA Investigators Endovascular thrombectomy for ischemic stroke increases disability-free survival, quality of life, and life expectancy and reduces cost. Front Neurol 2017 8 657 doi: 10.3389/fneur.2017.00657 29312109 10.3389/fneur.2017.00657 PMC5735082

[49] Hunter RM Davie C Rudd A Thompson A Walker H Thomson N Mountford J Schwamm L Deanfield J Thompson K Impact on clinical and cost outcomes of a centralized approach to acute stroke care in London: a comparative effectiveness before and after model. PLoS One 2013 8 e70420 doi: 10.1371/journal.pone.0070420 23936427 10.1371/journal.pone.0070420 PMC3731285

[50] Ramsay AI Morris S Hoffman A Hunter RM Boaden R McKevitt C Perry C Pursani N Rudd AG Turner SJ Effects of centralizing acute stroke services on stroke care provision in two large metropolitan areas in England. Stroke 2015 46 2244–2251 doi: 10.1161/STROKEAHA.115.009723 26130092 10.1161/STROKEAHA.115.009723 PMC4512749

[51] Kesteloot H Sans S Kromhout D Dynamics of cardiovascular and all-cause mortality in Western and Eastern Europe between 1970 and 2000. Eur Heart J 2006 27 107–113 doi: 10.1093/eurheartj/ehi511 16204263 10.1093/eurheartj/ehi511

[52] Wilkins E Wilson L Wickramasinghe K Bhatnagar P Leal J Luengo-Fernandez R European Cardiovascular Disease Statistics 2017 2017 Brussels, Belgium European Heart Network

