# Supplementary material for: Burden of Stroke in Europe: Thirty-Year Projections of Incidence, Prevalence, Deaths, and Disability-Adjusted Life Years
Source: Stroke. 2020 Jul 10;51(8):2418–27. doi: 10.1161/STROKEAHA.120.029606 (PMC7382540; doi:10.1161/STROKEAHA.120.029606)
Supplement: Supplementary file 1 [file str-51-2418-s001.pdf]

SUPPLEMENTAL MATERIAL

Burden of stroke in Europe: 30-year projections of incidence, prevalence, deaths, and disability-adjusted life years (DALYs)

Hatem A Wafa, Charles D. A. Wolfe, Eva Emmet, Gregory A Roth, Catherine O Johnson, and Yanzhong Wang

LIST OF TABLES

Table I: Models coefficients and goodness of fit in males. 4

Table II: Models coefficients and goodness of fit in females. 6

LIST OF FIGURES

Figure I: Prediction performance of different models in the UK..7

Figure II: Prediction performance of different models in Belgium. 7

Figure III: Projected number of incident strokes between 2017 and 2047. 8

Figure IV: Projected number of prevalent strokes between 2017 and 2047. 9

Figure V: Projected number of stroke deaths between 2017 and 2047. 10

Figure VI: Projected number of stroke DALYs between 2017 and 2047...............................11

Figure VII: Age- and sex-adjusted incidence rate of stroke in 2017 and 2047. 12

Figure VIII: Age- and sex-adjusted prevalence rate of stroke in 2017 and 2047. 13

Figure IX: Age- and sex-adjusted mortality rate of stroke in 2017 and 2047. 14

Figure X: Age- and sex-adjusted rate of DALYs lost because of stroke in 2017 and 2047....15

Age Group	Parameter	Incidence			Prevalence			Death			DALYs		
		Linear	*Poisson*	*Exponential*	*Linear*	*Poisson*	*Exponential*	*Linear*	*Poisson*	*Exponential*	*Linear*	*Poisson*	*Exponential*
**0-19**	**Years**	-0·569941*	-0·021427*	-0·019834*	-5·499448*	-0·026614*	-0·023579*	-0·067717*	-0·051268*	-0·056122*	-5·509371*	-0·035163*	-0·036223*
	**GDP**	2·6e-05*	1e-06*	2e-06*	0·001205*	6e-06*	6e-06*	-1·2e-05*	-1e-05*	-9e-06*	-0·001287*	-9e-06*	-8e-06*
	**AIC**	551·0242	Inf	156·929**	932·6114**	Inf	969·806	143·811**	Inf	731·875	812·4855	Inf	176·592**
**20-24**	**Years**	-0·053016*	-0·006402*	-0·006397*	-1·725499*	-0·014368*	-0·013784*	-0·044778*	-0·03732*	-0·042671*	-2·995584*	-0·028457*	-0·030882*
	**GDP**	-4·8e-05*	-7e-06*	-6e-06*	0·000182*	2e-06*	2e-06*	-1·6e-05*	-1·7e-05*	-1·3e-05*	-0·001272*	-1·5e-05*	-1·2e-05*
	**AIC**	245·4682	Inf	-784·318**	756·4926	Inf	378·741**	117·0295**	Inf	696·968	756·97	Inf	323·916**
**25-29**	**Years**	-0·062496*	-0·004199*	-0·004466*	-1·300165*	-0·007328*	-0·007061*	-0·06173*	-0·030436*	-0·036099*	-3·774259*	-0·023839*	-0·026832*
	**GDP**	-0·000122*	-1·1e-05*	-9e-06*	-0·000638*	-4e-06*	-3e-06*	-3·7e-05*	-2·6e-05*	-1·9e-05*	-0·002522*	-2·3e-05*	-1·6e-05*
	**AIC**	343·2588	Inf	-494·556**	771·7045	Inf	-122·202**	194·2332**	Inf	784·684	822·6137	Inf	454·601**
**30-34**	**Years**	-0·122258*	-0·004103*	-0·004721*	-1·305753*	-0·004602*	-0·004746*	-0·099894*	-0·022458*	-0·030939*	-5·553172*	-0·018422*	-0·023983*
	**GDP**	-0·000259*	-1·2e-05*	-1e-05*	-0·00192*	-8e-06*	-7e-06*	-9·3e-05*	-3·7e-05*	-2·6e-05*	-0·005714*	-3·3e-05*	-2·3e-05*
	**AIC**	446·9793	Inf	-468·255**	817·8775	Inf	-258·278**	295·726**	Inf	749·199	911·5821	Inf	505·304**
**35-39**	**Years**	-0·237246*	-0·00461*	-0·005502*	-1·589135*	-0·003185*	-0·003532*	-0·181236*	-0·018595*	-0·028785*	-9·115821*	-0·015495*	-0·022909*
	**GDP**	-0·000447*	-1·2e-05*	-1e-05*	-0·003584*	-9e-06*	-8e-06*	-0·000206*	-4·3e-05*	-2·8e-05*	-0·011443*	-3·9e-05*	-2·5e-05*
	**AIC**	540·7436	Inf	-397·082**	872·9555	Inf	-426·999**	418·7304**	Inf	920·377	1021·3376	Inf	745·984**
**40-44**	**Years**	-0·223551*	-0·001466*	-0·00282*	-1·08753	-0·000568*	-0·001307	-0·292984*	-0·010531*	-0·024341*	-13·239643*	-0·00886*	-0·019515*
	**GDP**	-0·001076*	-1·5e-05*	-1·3e-05*	-0·008263	-1·4e-05*	-0·000011	-0·000491*	-5·3e-05*	-3·3e-05*	-0·024264*	-4·8e-05*	-3e-05*
	**AIC**	650·9485	Inf	-255·651**	968·7138	Inf	-315·551**	538·0685**	Inf	1010·402	1125·2386	Inf	871·31**
**45-49**	**Years**	-0·07692	0·001065*	-0·000588	-0·378686	0·000846*	-0·000313	-0·418187*	-0·004748*	-0·019069*	-16·581917*	-0·003573*	-0·015039*
	**GDP**	-0·0021	-1·8e-05*	-0·000015	-0·015272	-1·5e-05*	-0·000012	-0·000962*	-5·6e-05*	-3·4e-05*	-0·042656*	-5·2e-05*	-3·2e-05*
	**AIC**	741·8795	Inf	-142·723**	1062·8467	Inf	-183·593**	632·7535**	Inf	1007·621	1203·9221	Inf	890·926**
**50-54**	**Years**	0·197291	0·002965*	0·000886	0·088305	0·001585*	0·000168	-0·631859*	-0·00103	-0·016011*	-21·874334*	-0·000165	-0·01228*
	**GDP**	-0·003895	-2·1e-05*	-0·000017	-0·028083	-1·8e-05*	-0·000014	-0·001818*	-0·00006	-3·6e-05*	-0·071815*	-0·000056	-3·4e-05*
	**AIC**	830·1925	Inf	-30·649**	1154·2813	Inf	-31·739**	729·4504**	Inf	1115·814	1283·2333	Inf	1001·639**
**55-59**	**Years**	1·139532*	0·005778*	0·003028*	6·191679	0·004115*	0·002054	-0·979391*	0·000835	-0·015554*	-28·18128*	0·002034*	-0·011429*
	**GDP**	-0·006421*	-2·4e-05*	-1·8e-05*	-0·05043	-2·1e-05*	-0·000016	-0·003222*	-0·000062	-3·7e-05*	-0·112446*	-5·8e-05*	-3·5e-05*
	**AIC**	904·1503	Inf	69·363**	1237·1123	Inf	107·692**	813·8462**	Inf	1129·73	1348·8309	Inf	1026·75**
**60-64**	**Years**	1·361238*	0·005413*	0·001816	13·865515*	0·00558*	0·003008*	-1·955617*	-0·001162*	-0·018078*	-47·502461*	0·000757*	-0·013555*
	**GDP**	-0·009466*	-2·5e-05*	-0·000018	-0·080572*	-2·3e-05*	-1·7e-05*	-0·005641*	-5·9e-05*	-3·6e-05*	-0·16979*	-5·6e-05*	-3·4e-05*
	**AIC**	963·16	Inf	114·434**	1303·0883	Inf	174·083**	898·0688**	Inf	1136·181	1410·5979	Inf	1046·304**
**65-69**	**Years**	1·600244	0·005046*	0·000535	27·401899*	0·006958*	0·003972*	-3·91492*	-0·004892*	-0·021514*	-78·990465*	-0·002202*	-0·01664*
	**GDP**	-0·012747	-2·5e-05*	-0·000018	-0·108524*	-2·3e-05*	-1·7e-05*	-0·009069*	-5·3e-05*	-3·3e-05*	-0·228703*	-5·1e-05*	-3·2e-05*
	**AIC**	1012·3594	Inf	173·563**	1345·1278	Inf	154·244**	971·2901**	Inf	1069·765	1456·3759	Inf	993·481**
**70-74**	**Years**	0·207662	0·00238*	-0·001857	34·29929*	0·006503*	0·003795*	-9·462104*	-0·012355*	-0·026071*	-161·391338*	-0·009123*	-0·020966*
	**GDP**	-0·015708	-2·2e-05*	-0·000017	-0·132244*	-2·2e-05*	-1·6e-05*	-0·014264*	-4·4e-05*	-2·9e-05*	-0·290212*	-4·3e-05*	-2·8e-05*
	**AIC**	1048·4037	Inf	100·505**	1374·6473	Inf	89·508**	1044·797	Inf	925·54**	1496·4453	Inf	843·985**

75-79	**Years**	-1·984921	-0·000007	-0·003717*	47·204485*	0·006452*	0·004143*	-18·394946*	-0·016973*	-0·027347*	-246·913826*	-0·013475*	-0·022439*
	**GDP**	-0·018191	-0·000018	-1·5e-05*	-0·140095*	-1·8e-05*	-1·4e-05*	-0·020463*	-3·4e-05*	-2·3e-05*	-0·324184*	-3·3e-05*	-2·3e-05*
	**AIC**	1080·4041	Inf	20·021**	1384·5808	Inf	-76·354**	1111·6524	Inf	762·605**	1523·3824	Inf	672·816**
80-84	**Years**	-6·408331*	-0·003023*	-0·005248*	83·075201*	0·008475*	0·006498*	-27·867657*	-0·016701*	-0·023963*	-274·453082*	-0·013294*	-0·019557*
	**GDP**	-0·018299*	-1·3e-05*	-1·1e-05*	-0·127202*	-1·4e-05*	-1·1e-05*	-0·027204*	-2·5e-05*	-1·8e-05*	-0·320264*	-2·5e-05*	-1·8e-05*
	**AIC**	1104·9504	Inf	-167·818**	1385·2735	Inf	-219·191**	1169·1972	Inf	573·75**	1534·1067	Inf	471·275**
85-89	**Years**	-15·083111*	-0·006752*	-0·0076*	116·266241*	0·010598*	0·009257*	-34·784979*	-0·014115*	-0·017968*	-237·796039*	-0·010987*	-0·014304*
	**GDP**	-0·016143*	-9e-06*	-7e-06*	-0·09886*	-1e-05*	-8e-06*	-0·032026*	-1·8e-05*	-1·4e-05*	-0·270441*	-1·8e-05*	-1·4e-05*
	**AIC**	1139·9675	Inf	-260·405**	1383·5937	Inf	-286·348**	1223·6051	Inf	402·992**	1535·6966	Inf	288·291**
90-94	**Years**	-19·89117*	-0·007747*	-0·008648*	142·555187*	0·013211*	0·012523*	-47·264285*	-0·014362*	-0·016607*	-215·94479*	-0·010976*	-0·012738*
	**GDP**	-0·013432*	-6e-06*	-5e-06*	-0·069496*	-7e-06*	-6e-06*	-0·032647*	-1·2e-05*	-1e-05*	-0·191217*	-1·2e-05*	-1e-05*
	**AIC**	1175·701	Inf	-181·122**	1407·5923	Inf	-67·242**	1263·2348	Inf	282·315**	1517·0982	Inf	148·502**
95+	**Years**	-4·840201	-0·001601*	-0·003458*	232·429596*	0·023252*	0·020672*	-14·132521	-0·002466*	-0·006302*	-13·327837	-0·000116*	-0·003366
	**GDP**	-0·012419	-5e-06*	-3e-06*	-0·056885*	-6e-06*	-3e-06*	-0·045688	-1·2e-05*	-9e-06*	-0·159479	-1·2e-05*	-0·000008
	**AIC**	1260·9626	Inf	446·471**	1489·264	Inf	678·162**	1366·3943	Inf	773·812**	1549·6182	Inf	667·476**

Table I: Models coefficients and goodness of fit based on the AIC criteria for each stroke measure by age groups in males.

* denotes significant coefficient (p<0·05).
** indicates the selected model (lowest AIC).

Abbreviations: AIC, Akaike information criterion; and GDP, gross domestic product per capita adjusted for purchasing power parity in 2011 US Dollars.

Age Group	Parameter	Incidence			Prevalence			Death			DALYs		
		Model 1	***Model 2***	***Model 3***	***Model 1***	***Model 2***	***Model 3***	***Model 1***	***Model 2***	***Model 3***	***Model 1***	***Model 2***	***Model 3***
**0-19**	**Years**	-0·333107*	-0·011659*	-0·011018*	-2·537525*	-0·012284*	-0·010465*	-0·051215*	-0·049019*	-0·052263*	-3·83362*	-0·025722*	-0·026004*
	**GDP**	-8·7e-05*	-3e-06*	-2e-06*	0·00026*	1e-06*	2e-06*	-4e-06*	-4e-06*	-3e-06*	-0·000923*	-7e-06*	-6e-06*
	**AIC**	527·4975	Inf	-101·752**	887·5231	Inf	777·472**	108·7079**	Inf	841·917	784·0906	Inf	45·98**
**20-24**	**Years**	-0·012107*	-0·001021	-0·001329*	-0·019087	0·000181	0·000107	-0·032003*	-0·036438*	-0·040967*	-1·985734*	-0·021608*	-0·023194*
	**GDP**	-6·3e-05*	-0·000008	-7e-06*	-0·00094	-0·000008	-0·000006	-1e-05*	-1·3e-05*	-1e-05*	-0·000957*	-1·3e-05*	-1e-05*
	**AIC**	243·5325	Inf	-906·509**	741·415	Inf	16·091**	45·5829**	Inf	583·825	697·3852	Inf	7·045**
**25-29**	**Years**	-0·003475	0·00027	-0·000102	0·07088	0·000635	0·000433	-0·05027*	-0·037117*	-0·043028*	-2·847673*	-0·022431*	-0·025061*
	**GDP**	-0·000126	-0·00001	-0·000008	-0·001228	-0·000007	-0·000006	-1·5e-05*	-1·3e-05*	-9e-06*	-0·001376*	-1·3e-05*	-1e-05*
	**AIC**	334·1322	Inf	-750·617**	762·5498	Inf	-288·446**	104·6761**	Inf	489·445	747·7216	Inf	-2·738**
**30-34**	**Years**	-0·091098*	-0·003259*	-0·003463*	0·38743	0·001694*	0·001308	-0·079373*	-0·033346*	-0·038747*	-4·116911*	-0·021017*	-0·023831*
	**GDP**	-0·000169*	-7e-06*	-6e-06*	-0·002332	-9e-06*	-0·000007	-3·2e-05*	-1·7e-05*	-1·3e-05*	-0·002591*	-1·7e-05*	-1·3e-05*
	**AIC**	401·0426	Inf	-920·86**	812·4511	Inf	-417·603**	205·1058**	Inf	609·678	832·8022	Inf	184·807**
**35-39**	**Years**	-0·283276*	-0·006659*	-0·007013*	0·221467	0·000943*	0·000581	-0·150417*	-0·031882*	-0·038992*	-7·268494*	-0·021842*	-0·025922*
	**GDP**	-0·000176*	-5e-06*	-4e-06*	-0·00398	-9e-06*	-0·000008	-7·3e-05*	-2·1e-05*	-1·4e-05*	-0·004916*	-2e-05*	-1·4e-05*
	**AIC**	485·2766	Inf	-733·151**	864·7858	Inf	-668·988**	306·4725**	Inf	586·293	920·3755	Inf	276·613**
**40-44**	**Years**	-0·366346*	-0·004883*	-0·005628*	-0·287851	0·000101	-0·000293	-0·242791*	-0·024608*	-0·032446*	-10·64093*	-0·017522*	-0·02251*
	**GDP**	-0·000511*	-8e-06*	-7e-06*	-0·006228	-0·00001	-0·000008	-0·000188*	-3e-05*	-2e-05*	-0·010505*	-2·8e-05*	-1·9e-05*
	**AIC**	597·0921	Inf	-409·4**	934·5924	Inf	-650·831**	413·5503**	Inf	564·268	1011·2821	Inf	342·342**
**45-49**	**Years**	-0·366651*	-0·0026*	-0·003775*	-0·477491	0·000192	-0·000418	-0·373927*	-0·018722*	-0·028045*	-14·578784*	-0·013212*	-0·019559*
	**GDP**	-0·001132*	-1·2e-05*	-1e-05*	-0·01069	-0·000011	-0·000009	-0·000402*	-3·7e-05*	-2·3e-05*	-0·019647*	-3·4e-05*	-2·2e-05*
	**AIC**	694·9057	Inf	-166·532**	1019·6916	Inf	-458·428**	517·7485**	Inf	688·612	1097·8991	Inf	513·879**
**50-54**	**Years**	-0·400247*	-0·001208*	-0·002894*	-1·550938	-0·000038	-0·000908	-0·554508*	-0·013837*	-0·023869*	-19·074905*	-0·009285*	-0·016248*
	**GDP**	-0·002127*	-1·6e-05*	-1·3e-05*	-0·019008	-0·000013	-0·00001	-0·000784*	-4·6e-05*	-2·7e-05*	-0·033859*	-4·1e-05*	-2·5e-05*
	**AIC**	770·6347	Inf	-38·587**	1108·4757	Inf	-238·063**	617·4984**	Inf	831·042	1179·7727	Inf	679·122**
**55-59**	**Years**	-0·185381	0·001006*	-0·001485	-0·374178	0·001087*	-0·000363	-0·906354*	-0·011774*	-0·023915*	-26·616641*	-0·007088*	-0·015745*
	**GDP**	-0·00346	-2e-05*	-0·000016	-0·033126	-1·6e-05*	-0·000012	-0·001433*	-5·3e-05*	-3e-05*	-0·054458*	-4·7e-05*	-2·8e-05*
	**AIC**	831·9758	Inf	56·838**	1190·3113	Inf	-40·708**	707·336**	Inf	984·69	1250·7626	Inf	832·807**
**60-64**	**Years**	-0·096599	0·002049*	-0·000917	1·076533	0·001961*	-0·000122	-1·822079*	-0·014214*	-0·027678*	-45·729131*	-0·008706*	-0·018765*
	**GDP**	-0·005614	-2·3e-05*	-0·000017	-0·053772	-1·9e-05*	-0·000014	-0·002592*	-5·3e-05*	-3e-05*	-0·084553*	-4·8e-05*	-2·9e-05*
	**AIC**	893·6774	Inf	107·241**	1259·6859	Inf	97·46**	799·517**	Inf	1096·266	1319·6999	Inf	949·764**
**65-69**	**Years**	0·275382	0·003271*	-0·000063	9·546429	0·004091*	0·00132	-3·841461*	-0·016364*	-0·031565*	-79·860655*	-0·010472*	-0·022427*
	**GDP**	-0·008453	-2·4e-05*	-0·000018	-0·081081	-2·1e-05*	-0·000016	-0·005001*	-5·2e-05*	-3·1e-05*	-0·133992*	-4·9e-05*	-2·9e-05*
	**AIC**	951·3383	Inf	172·622**	1313·1884	Inf	187·319**	895·1313**	Inf	1094·388	1385·7901	Inf	971·709**
**70-74**	**Years**	-0·116798	0·002436*	-0·001328	20·275989*	0·005616*	0·002629*	-8·765941*	-0·020309*	-0·034421*	-149·732809*	-0·014579*	-0·026009*
	**GDP**	-0·012742	-2·4e-05*	-0·000018	-0·112791*	-2·3e-05*	-1·7e-05*	-0·009541*	-4·5e-05*	-2·8e-05*	-0·202503*	-4·4e-05*	-2·7e-05*
	**AIC**	1007·7853	Inf	140·071**	1355·0623	Inf	224·75**	994·5277**	Inf	1028·922	1449·3022	Inf	920·402**

**75-79**	**Years**	-0·98649	0·001507*	-0·002828*	41·058041*	0·007383*	0·004415*	-16·740892*	-0·01957*	-0·032219*	-222·25333*	-0·014798*	-0·025258*
	**GDP**	-0·0185	-2·3e-05*	-1·8e-05*	-0·141559*	-2·3e-05*	-1·7e-05*	-0·017705*	-3·9e-05*	-2·5e-05*	-0·286032*	-3·8e-05*	-2·5e-05*
	**AIC**	1067·1209	Inf	169·632**	1384·3943	Inf	177·325**	1085·8789	Inf	882·276**	1499·6231	Inf	781·471**
**80-84**	**Years**	-4·4926*	-0·001547*	-0·004349*	82·848716*	0·010245*	0·007567*	-25·642046*	-0·01604*	-0·025409*	-246·556231*	-0·01208*	-0·02012*
	**GDP**	-0·021*	-1·7e-05*	-1·4e-05*	-0·152068*	-1·9e-05*	-1·5e-05*	-0·028542*	-3·1e-05*	-2·1e-05*	-0·340289*	-3e-05*	-2·1e-05*
	**AIC**	1098·3412	Inf	-84·942**	1396·4612	Inf	45·998**	1163·1378	Inf	694·503**	1530·0356	Inf	601·981**
**85-89**	**Years**	-12·053649*	-0·005135*	-0·006498*	118·181585*	0·012048*	0·010389*	-31·266005*	-0·012001*	-0·017612*	-205·667088*	-0·008734*	-0·013594*
	**GDP**	-0·020506*	-1·2e-05*	-1e-05*	-0·133862*	-1·5e-05*	-1·2e-05*	-0·037598*	-2·2e-05*	-1·6e-05*	-0·320692*	-2·2e-05*	-1·6e-05*
	**AIC**	1132·9152	Inf	-286·482**	1393·9186	Inf	-116·766**	1222·8373	Inf	448·368**	1537·4938	Inf	357·571**
**90-94**	**Years**	-18·149367*	-0·006755*	-0·007912*	137·246856*	0·013572*	0·012705*	-39·408615*	-0·010657*	-0·014276*	-170·511348*	-0·007541*	-0·010532*
	**GDP**	-0·019773*	-9e-06*	-7e-06*	-0·103192*	-1·1e-05*	-9e-06*	-0·0449*	-1·7e-05*	-1·3e-05*	-0·262715*	-1·7e-05*	-1·3e-05*
	**AIC**	1152·7056	Inf	-402·471**	1388·9202	Inf	-162·056**	1270·4505	Inf	281·495**	1526·5578	Inf	178·986**
**95+**	**Years**	-15·220984*	-0·005016*	-0·006024*	174·132807*	0·01837*	0·016856*	-23·493619*	-0·003998*	-0·007044*	-43·465139	-0·001548*	-0·004225*
	**GDP**	-0·020062*	-8e-06*	-7e-06*	-0·083201*	-9e-06*	-8e-06*	-0·056322*	-1·5e-05*	-1·1e-05*	-0·2027	-1·5e-05*	-1·2e-05*
	**AIC**	1200·1054	Inf	-27·87**	1424·6332	Inf	311·252**	1328·5702	Inf	383·322**	1512·7313	Inf	319·679**

Table II: Models coefficients and goodness of fit based on the AIC criteria for each stroke measure by age groups in females.

* denotes significant coefficient ($p<0·05$).
** indicates the selected model (lowest AIC).

Abbreviations: AIC, Akaike information criterion; and GDP, gross domestic product per capita adjusted for purchasing power parity in 2011 US Dollars.

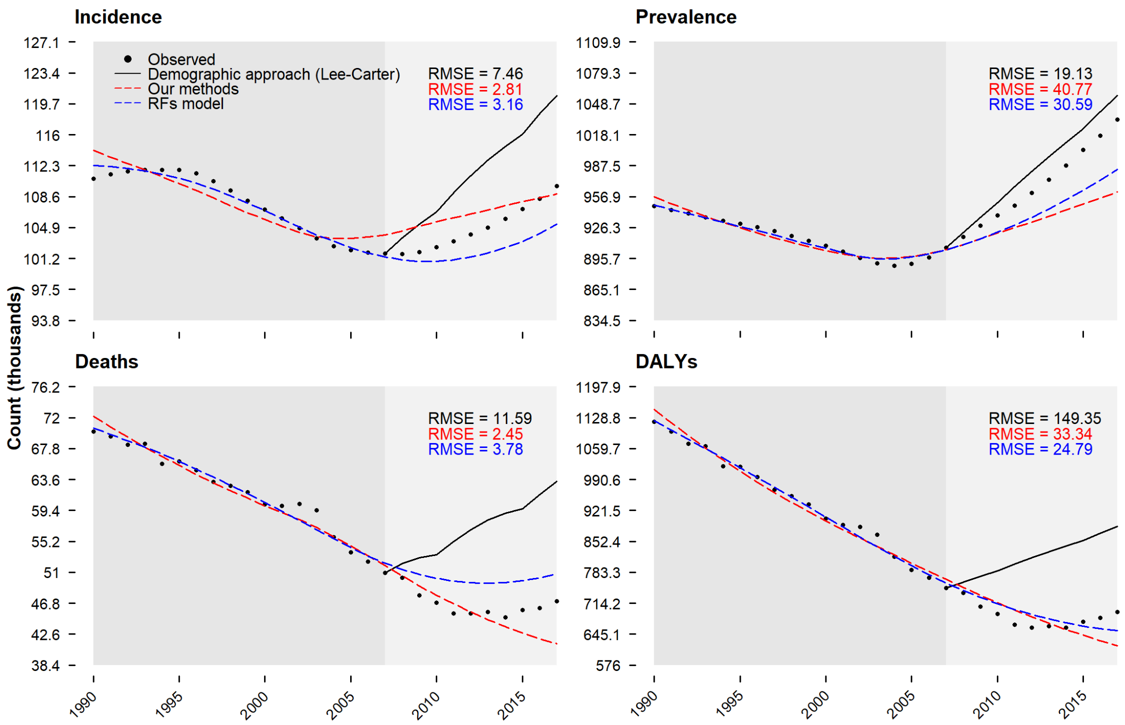

Figure I: Prediction performance of different models in the UK.

Demographic approach assumes constant age-specific rates and apply those to the corresponding populations in each prediction year. RFs model is a regression model adjusted for the changes in prevalence of hypertension, diabetes mellitus, and atrial fibrillation.

Abbreviations: RFs, risk factors; RMSE, root mean squared error; and DALYs, disability-adjusted life years.

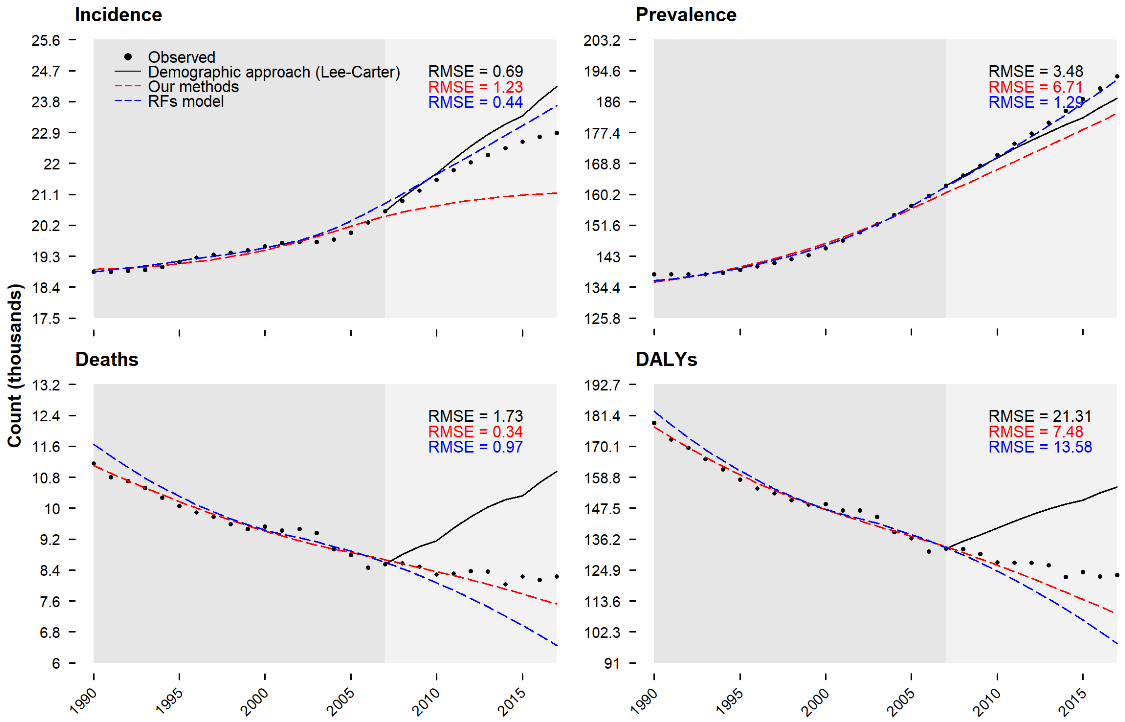

Figure II: Prediction performance of different models in Belgium.

Demographic approach assumes constant age-specific rates and apply those to the corresponding populations in each prediction year. RFs model is a regression model adjusted for the changes in prevalence of hypertension, diabetes mellitus, and atrial fibrillation.

Abbreviations: RFs, risk factors; RMSE, root mean squared error; and DALYs, disability-adjusted life years.

Country	2017	2027	2047
**1 Cyprus**	1·73 (1·68-1·79)	2·07 (1·97-2·17)	2·76 (2·5-3·06)
**2 Ireland**	6·2 (5·9-6·4)	6·9 (6·5-7·4)	8·3 (7·1-9·8)
**3 Poland**	98 (94-102)	111 (102-121)	129 (107-172)
**4 Luxembourg**	0·84 (0·8-0·87)	0·9 (0·85-0·96)	1·09 (0·97-1·24)
**5 Belgium**	22·9 (22·4-23·5)	24·6 (23·8-25·3)	29·3 (27·4-31·3)
**6 Lithuania**	12 (11·6-12·4)	13·1 (12·4-13·8)	15·2 (13·7-16·8)
**7 Finland**	15·2 (14·7-15·7)	17·7 (16·9-18·5)	19·2 (17·7-21)
**8 Slovakia**	15·4 (14·9-15·9)	17·6 (16·4-18·8)	18·8 (15·4-23·2)
**9 Malta**	0·75 (0·72-0·79)	0·84 (0·78-0·9)	0·86 (0·74-1)
**10 Sweden**	19·1 (18·5-19·7)	20·2 (19·3-21·2)	21·7 (19·8-23·7)
**11 Austria**	20·7 (19·7-21·7)	22·2 (20·8-23·6)	23·2 (20·8-25·8)
**12 Netherlands**	32·9 (32·2-33·5)	35·3 (34·2-36·4)	36·1 (33·9-38·6)
**13 Romania**	79 (76-81)	82 (78-86)	86 (73-101)
**14 France**	112 (108-115)	113 (107-120)	119 (105-135)
**15 Denmark**	11 (10·8-11·2)	11·6 (11·3-12)	11·4 (10·6-12·2)
**16 Germany**	189 (181-198)	194 (180-208)	194 (167-226)
**17 Croatia**	13·9 (13·3-14·6)	14 (13·2-15)	13·9 (12·3-15·6)
**18 United Kingdom**	106 (103-110)	107 (102-112)	104 (96-114)
**19 Czech Republic**	29·1 (28-30·2)	30·3 (28·3-32·6)	27·9 (24·6-31·7)
**20 Spain**	90 (88-92)	91 (87-94)	86 (81-92)
**21 Slovenia**	4·2 (4·04-4·36)	4·23 (3·96-4·51)	3·98 (3·51-4·54)
**22 Bulgaria**	29·3 (28·5-30)	28·8 (27·6-30·1)	27·3 (24·8-30·1)
**23 Greece**	28·6 (27·7-29·6)	27·3 (26-28·7)	25·9 (23·6-28·7)
**24 Latvia**	8·5 (8·1-8·9)	8·2 (7·6-8·8)	7·7 (6·7-8·8)
**25 Italy**	117·5 (112·9-122·4)	112·2 (105·2-119·6)	100·6 (90·2-112·5)
**26 Hungary**	26·8 (25·9-27·8)	26·2 (24·6-27·9)	22·5 (19·9-25·6)
**27 Estonia**	3·59 (3·45-3·74)	3·33 (3·11-3·56)	2·75 (2·36-3·22)
**28 Portugal**	22·4 (21·8-23·1)	20·3 (19·3-21·4)	17·3 (15·1-20)

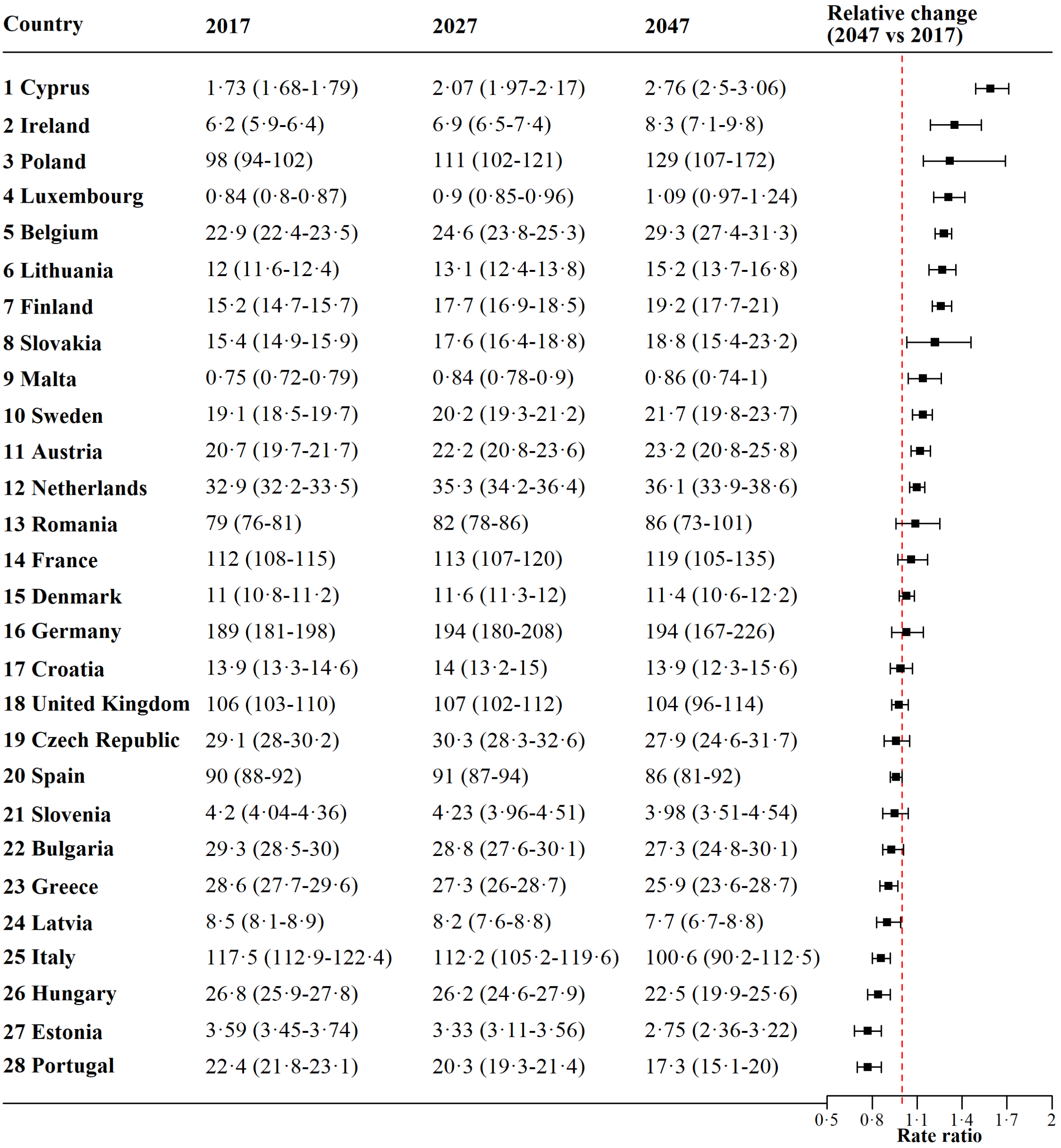

Figure III: Projected number of incident strokes (in thousands) between 2017 and 2047 in European countries.

Data are counts in thousands (95% CI) as obtained from models.

Country	2017	2027	2047
**1 Cyprus**	14·72 (14·29-15·15)	18·18 (17·4-19·01)	25·71 (23·55-28·09)
**2 Luxembourg**	7·79 (7·51-8·08)	9·19 (8·66-9·75)	13·2 (11·74-14·85)
**3 Austria**	171·2 (162·4-180·4)	205·3 (191·2-220·3)	278·5 (246·2-313·5)
**4 Finland**	127·7 (124·3-131·2)	157·8 (151·7-164·2)	208 (191·9-226·1)
**5 Ireland**	59·9 (57·8-62)	71·1 (67·5-74·9)	95·1 (85-106·1)
**6 Belgium**	193·5 (189·2-197·9)	221·3 (214·3-228·3)	298 (280·3-316·3)
**7 Slovakia**	130·9 (127·5-134·4)	158·5 (149·9-167·7)	201·5 (168·3-244·5)
**8 Poland**	804 (763-848)	924 (780-1100)	1156 (693-2236)
**9 Germany**	1695 (1625-1769)	1906 (1779-2041)	2380 (2082-2713)
**10 Spain**	788 (772-803)	883 (855-911)	1089 (1028-1154)
**11 France**	1009 (981-1038)	1128 (1074-1185)	1388 (1252-1537)
**12 Netherlands**	293·1 (289·1-297·3)	339·2 (332·1-346·5)	397·4 (379·3-417·1)
**13 Lithuania**	103·8 (101·1-106·5)	115·4 (110·8-120)	138·4 (129-148)
**14 Sweden**	190·7 (185·6-195·9)	211·5 (203·5-219·8)	249·8 (231·9-269·6)
**15 Malta**	6·34 (6·07-6·63)	7·2 (6·69-7·76)	7·87 (6·78-9·19)
**16 Denmark**	98·2 (96·3-100·1)	109·3 (106·5-112·2)	120·4 (113·7-127·8)
**17 Slovenia**	36·83 (35·14-38·6)	39·47 (36·52-42·69)	44·04 (37·8-51·55)
**18 Croatia**	113·1 (108·2-118·3)	118·4 (110·8-126·6)	133·2 (117·2-151·5)
**19 United Kingdom**	1009 (986-1033)	1072 (1037-1110)	1189 (1109-1280)
**20 Greece**	208·1 (202·4-214)	212·9 (204·7-221·7)	234·7 (217·4-254·7)
**21 Bulgaria**	256·2 (249·8-262·8)	269·6 (258·5-280·9)	284·9 (263·6-307)
**22 Czech Republic**	249 (239·1-259·4)	257·2 (238·7-277·4)	266·1 (233·7-304·9)
**23 Romania**	629 (609-649)	653 (619-688)	662 (563-784)
**24 Latvia**	73·8 (70·6-77·2)	73·5 (68·8-78·6)	73 (64·4-82·7)
**25 Hungary**	263·1 (253·2-273·5)	268·7 (251·2-287·6)	256·5 (225-293)
**26 Italy**	768 (739·3-797·9)	757·8 (712·7-805·9)	734·2 (655·1-824)
**27 Portugal**	192·3 (188·7-196)	183·1 (177·3-189·1)	159·1 (145-175)
**28 Estonia**	34·55 (33·2-35·95)	31·77 (29·67-34·04)	28·34 (24·04-33·53)

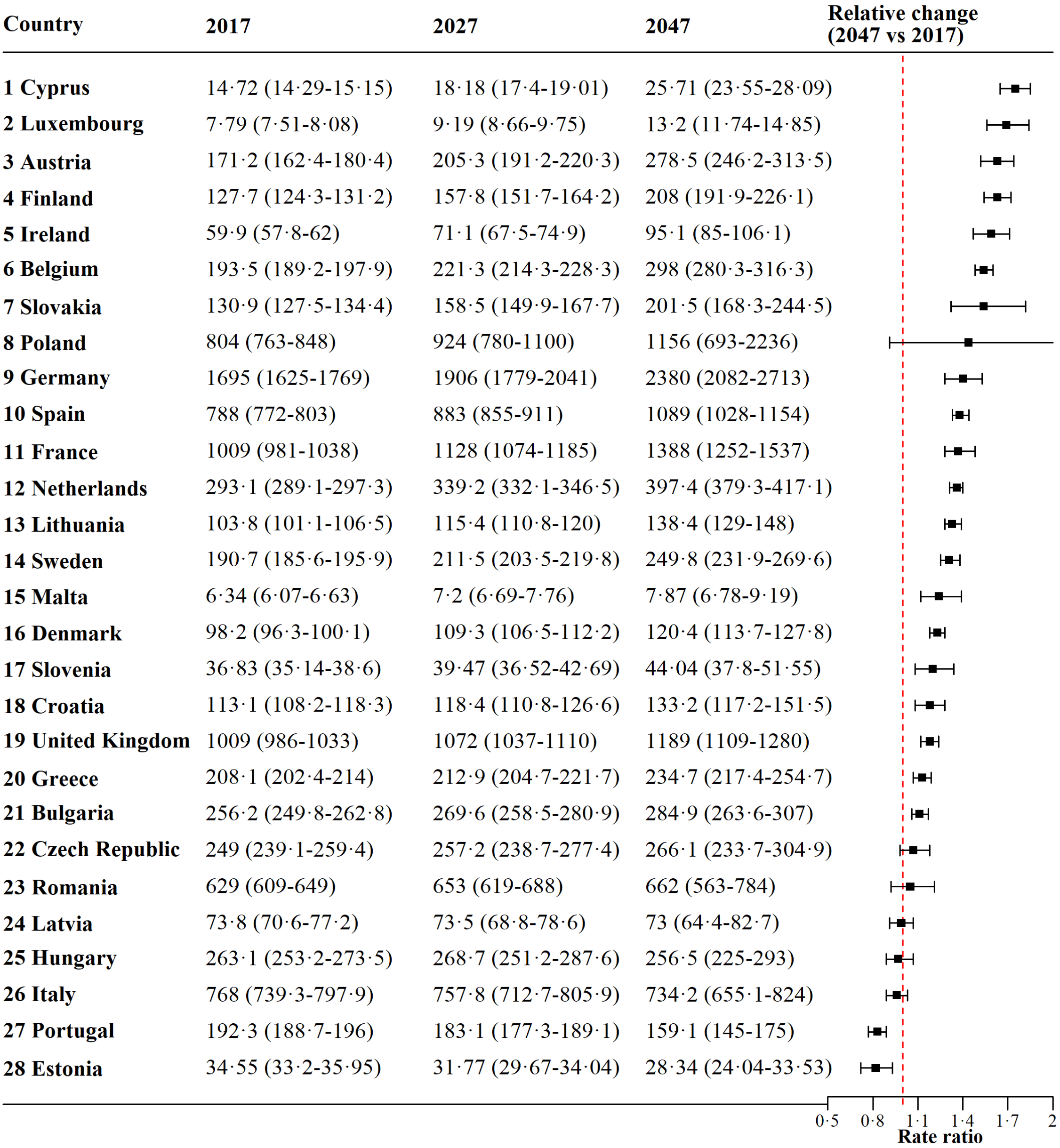

Figure IV: Projected number of prevalent strokes (in thousands) between 2017 and 2047 in European countries.

Data are counts in thousands (95% CI) as obtained from models.

Country	2017	2027	2047
1 Cyprus	0·56 (0·52-0·6)	0·62 (0·57-0·69)	0·86 (0·72-1·03)
2 Lithuania	5·5 (5·1-5·9)	6 (5·3-6·7)	7·4 (6-9)
3 Ireland	2 (1·9-2·1)	2 (1·9-2·2)	2·6 (2·1-3·4)
4 Malta	0·31 (0·29-0·33)	0·35 (0·32-0·38)	0·39 (0·33-0·47)
5 Romania	51 (48-54)	52 (47-57)	65 (49-87)
6 Netherlands	10·1 (9·6-10·7)	10·4 (9·5-11·3)	10·6 (9·1-12·5)
7 Sweden	7·6 (7·3-7·9)	7·8 (7·3-8·2)	8 (7·1-9)
8 Denmark	3·9 (3·7-4·1)	3·9 (3·6-4·1)	3·9 (3·4-4·6)
9 Bulgaria	21·9 (21-22·9)	21·3 (19·7-22·9)	21·9 (18·5-26·1)
10 Slovenia	1·8 (1·71-1·91)	1·68 (1·52-1·86)	1·74 (1·41-2·19)
11 Latvia	4·6 (4·3-5·1)	4·2 (3·6-4·9)	4·4 (3·1-6·4)
12 United Kingdom	44 (43-46)	42 (39-45)	40 (36-46)
13 Italy	58 (55·9-60·2)	55·2 (51·9-58·8)	51·8 (45·9-58·5)
14 Finland	4·3 (4·2-4·5)	4·1 (3·8-4·3)	3·8 (3·4-4·3)
15 Slovakia	5·2 (5-5·5)	5·1 (4·6-5·7)	4·6 (3·2-6·9)
16 Belgium	8·1 (7·8-8·4)	7·1 (6·7-7·5)	6·8 (6-7·6)
17 Croatia	6·9 (6·5-7·4)	6·1 (5·6-6·8)	5·7 (4·8-6·9)
18 Luxembourg	0·32 (0·3-0·33)	0·27 (0·25-0·29)	0·25 (0·21-0·31)
19 Greece	15·3 (14·2-16·5)	14·1 (12·5-16)	12 (9·6-15·6)
20 France	38 (36-40)	32 (29-35)	28 (22-35)
21 Poland	40 (38-44)	34 (26-44)	27 (11-101)
22 Spain	31 (30-32)	26 (25-27)	20 (18-22)
23 Germany	59 (56-63)	50 (45-56)	35 (28-48)
24 Hungary	11·7 (11·2-12·3)	9·8 (9-10·6)	6·8 (5·7-8·1)
25 Portugal	13·6 (12·9-14·3)	10·9 (10·1-11·9)	7·3 (5·9-9·1)
26 Czech Republic	10·2 (9·5-11)	8·2 (7·1-9·4)	5·3 (4·1-6·9)
27 Austria	4·5 (4·2-4·8)	3·3 (3-3·6)	1·9 (1·6-2·2)
28 Estonia	1·05 (0·93-1·18)	0·63 (0·52-0·77)	0·23 (0·15-0·35)

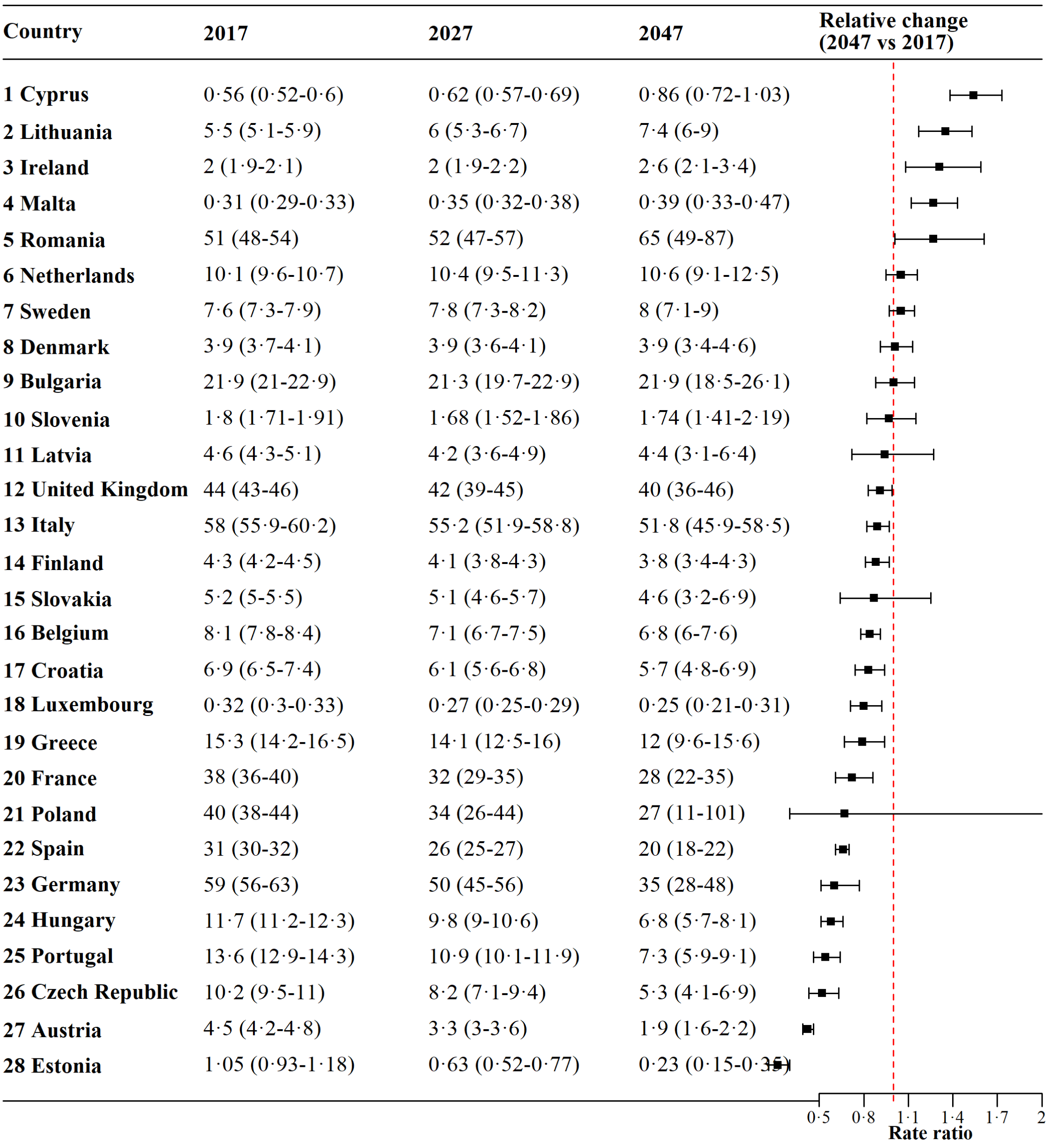

Figure V: Projected number of stroke deaths (in thousands) between 2017 and 2047 in European countries.

Data are counts in thousands (95% CI) as obtained from models.

Country	2017	2027	2047	Relative change (2047 vs 2017)
**1 Romania**	848 (801-899)	834 (762-913)	967 (735-1281)	
**2 Lithuania**	94·2 (89·2-99·6)	94·3 (86·2-103·7)	103·1 (84·1-133)	
**3 Cyprus**	9·34 (8·82-9·9)	9·42 (8·66-10·27)	9·73 (8·33-11·42)	
**4 Malta**	4·65 (4·39-4·92)	4·7 (4·31-5·12)	3·99 (3·4-4·7)	
**5 Sweden**	108·5 (105·2-112)	105·7 (101-110·6)	90·7 (82·9-99·5)	
**6 Ireland**	32·3 (30·7-33·9)	29·7 (27·5-32·1)	26·9 (21·8-33·4)	
**7 Netherlands**	161·2 (153·2-169·6)	157·4 (146·1-169·6)	133·1 (115·6-153·3)	
**8 Denmark**	60·2 (58-62·5)	55·9 (53-58·9)	44·9 (39·8-50·7)	
**9 Bulgaria**	364·4 (352·3-377·1)	326 (307·6-345·9)	269·8 (236·5-310·4)	
**10 Slovakia**	105·9 (102·1-109·9)	101 (93·2-109·5)	78·5 (60·3-104·4)	
**11 Belgium**	119·9 (116·2-123·7)	104·4 (100·1-109)	86·2 (78·9-94·3)	
**12 Finland**	69·6 (67·6-71·6)	62·8 (60·3-65·4)	47·5 (43·7-51·9)	
**13 United Kingdom**	637 (616-659)	559 (532-587)	432 (394-476)	
**14 Croatia**	113·2 (106·8-120)	94·7 (86·7-103·4)	73·9 (63·1-86·8)	
**15 Greece**	199·3 (188·7-210·7)	170·2 (156·8-185·3)	129·9 (110·5-155)	
**16 Luxembourg**	4·73 (4·51-4·96)	3·9 (3·61-4·22)	3·05 (2·62-3·57)	
**17 Italy**	675·1 (651·8-699·3)	568·6 (537·4-601·8)	427 (385·8-473·5)	
**18 Slovenia**	28·01 (26·7-29·39)	23·19 (21·41-25·15)	17·44 (14·85-20·63)	
**19 France**	558 (536-581)	467 (435-501)	342 (293-402)	
**20 Latvia**	76·9 (71·4-82·9)	62 (55·4-69·5)	44·5 (35·3-57·2)	
**21 Spain**	437 (425-450)	356 (340-373)	245 (226-266)	
**22 Germany**	933 (890-978)	764 (707-827)	505 (431-611)	
**23 Poland**	750 (709-794)	611 (503-745)	382 (202-897)	
**24 Hungary**	220·5 (211·9-229·4)	176·3 (165·2-188·2)	104·8 (92·5-119·1)	
**25 Austria**	76·7 (72·7-81)	58·5 (54·6-62·8)	33·4 (29·7-37·7)	
**26 Czech Republic**	173·9 (165·8-182·5)	133·2 (121·8-146·1)	73·4 (62·1-87·5)	
**27 Portugal**	178 (170·1-186·2)	127·7 (119-137·2)	66·2 (55·2-79·5)	
**28 Estonia**	21·3 (19·23-23·61)	12·75 (10·77-15·11)	4·48 (3·14-6·42)	

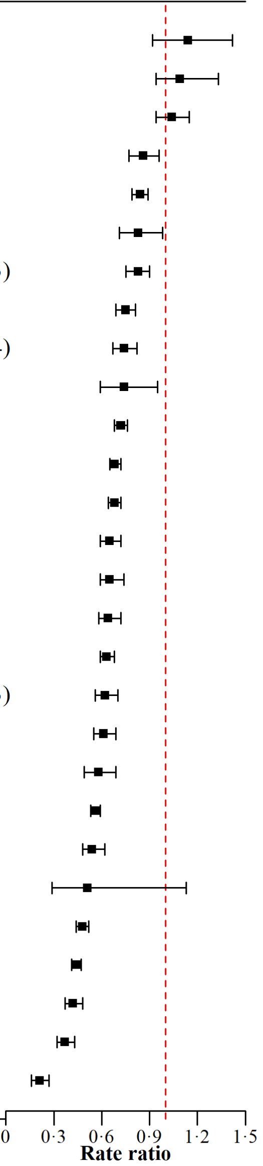

Figure VI: Projected number of stroke DALYs (in thousands) between 2017 and 2047 in EU countries.

Data are counts in thousands (95% CI) as obtained from models.

2017		2047		2047 vs 2017
Age-adjusted rate* (95% CI)	Country	Country	Age-adjusted rate* (95% CI)	% Change (95% CI)
428 (414-442)	1 Romania	1 Lithuania	467 (425-512)	14·4 (7·7 to 21·3)
421 (402-442)	2 Latvia	2 Romania	382 (326-450)	-10·6 (-21·2 to 1·9)
408 (395-422)	3 Lithuania	3 Latvia	368 (323-421)	-12·6 (-19·5 to -4·7)
406 (396-417)	4 Bulgaria	4 Bulgaria	356 (324-391)	-12·5 (-18 to -6·4)
348 (336-359)	5 Slovakia	5 Slovakia	278 (230-342)	-19·9 (-31·6 to -4·8)
326 (313-341)	6 Croatia	6 Croatia	270 (240-303)	-17·3 (-23·1 to -11)
293 (281-305)	7 Czech Republic	7 Poland	255 (214-327)	-12·1 (-23·1 to 8·5)
290 (279-301)	8 Poland	8 Finland	244 (223-266)	-7·9 (-12·6 to -2·9)
290 (279-301)	9 Hungary	9 Hungary	199 (177-224)	-31·3 (-36·4 to -25·6)
271 (260-282)	10 Estonia	10 Czech Republic	196 (175-221)	-33 (-37·9 to -27·4)
265 (256-274)	11 Finland	11 Austria	191 (172-213)	-17·5 (-22·2 to -12·4)
232 (225-239)	12 Greece	12 Estonia	181 (157-211)	-33 (-39·8 to -25·3)
232 (221-243)	13 Austria	13 Belgium	164 (155-175)	-17·9 (-20·9 to -14·7)
204 (195-213)	14 Germany	14 Germany	160 (140-184)	-21·5 (-28·2 to -13·8)
201 (195-208)	15 Cyprus	15 Cyprus	159 (145-175)	-21·1 (-25·5 to -16·2)
200 (196-205)	16 Belgium	16 Sweden	154 (141-168)	-20·2 (-24·5 to -15·5)
200 (196-204)	17 Netherlands	17 Greece	151 (139-164)	-35 (-38·1 to -31·4)
200 (192-208)	18 Slovenia	18 Netherlands	148 (139-157)	-26·1 (-28·8 to -23·3)
199 (195-203)	19 Denmark	19 Denmark	144 (135-155)	-27·5 (-30·9 to -23·9)
194 (188-200)	20 Portugal	20 Malta	132 (115-152)	-30·5 (-36·5 to -23·8)
193 (187-199)	21 Sweden	21 France	127 (115-142)	-23 (-28·6 to -16·7)
190 (182-199)	22 Malta	22 Slovenia	121 (108-137)	-39·4 (-43·9 to -34·3)
182 (177-186)	23 Spain	23 Ireland	119 (102-140)	-30·2 (-37·9 to -21·1)
174 (168-181)	24 Luxembourg	24 Spain	119 (112-127)	-34·3 (-36·9 to -31·6)
171 (165-178)	25 Ireland	25 Luxembourg	119 (105-134)	-32 (-37·2 to -26·1)
166 (161-171)	26 United Kingdom	26 United Kingdom	108 (100-116)	-35 (-37·9 to -31·8)
165 (161-170)	27 France	27 Portugal	103 (91-117)	-47·2 (-51·9 to -41·7)
162 (156-169)	28 Italy	28 Italy	92 (82-104)	-43·1 (-47·1 to -38·8)

Figure VII: Age- and sex-adjusted incidence rate of stroke (per 100,000) in 2017 and 2047.

* adjusted to the 2013 European Standard Population.

2017		2047		2047 vs 2017
Age-adjusted rate* (95% CI)	Country	Country	Age-adjusted rate* (95% CI)	% Change (95% CI)
367 (351-384)	1 Latvia	1 Lithuania	428 (401-456)	21 (16·3 to 25·7)
354 (345-363)	2 Lithuania	2 Bulgaria	385 (357-414)	11·1 (5·7 to 16·5)
346 (337-356)	3 Bulgaria	3 Latvia	353 (314-397)	-3·8 (-10·6 to 3·6)
336 (325-348)	4 Romania	4 Slovakia	301 (254-363)	3·9 (-10·1 to 21·5)
290 (282-298)	5 Slovakia	5 Romania	300 (257-353)	-10·7 (-21·1 to 1·7)
279 (268-291)	6 Hungary	6 Croatia	258 (228-292)	-2·1 (-9·4 to 5·9)
264 (252-276)	7 Croatia	7 Finland	258 (240-278)	16·1 (10·9 to 21·7)
261 (250-271)	8 Estonia	8 Poland	234 (141-430)	0·2 (-36·2 to 74·7)
246 (236-257)	9 Czech Republic	9 Hungary	227 (201-258)	-18·7 (-25·2 to -11·5)
233 (221-246)	10 Poland	10 Austria	211 (187-237)	9·8 (2·7 to 17·2)
222 (216-228)	11 Finland	11 Germany	187 (165-212)	1·8 (-6·3 to 10·4)
194 (189-199)	12 Sweden	12 Czech Republic	185 (163-211)	-24·8 (-30·8 to -17·9)
192 (182-202)	13 Austria	13 Sweden	181 (168-196)	-6·4 (-10·8 to -1·8)
184 (176-192)	14 Germany	14 Belgium	172 (163-182)	0·8 (-2·7 to 4·2)
177 (174-179)	15 Netherlands	15 Estonia	170 (146-199)	-34·7 (-41·7 to -26·6)
175 (172-179)	16 Denmark	16 Netherlands	163 (156-170)	-7·7 (-10·2 to -5)
174 (166-183)	17 Slovenia	17 Denmark	155 (146-165)	-11·6 (-14·9 to -8)
174 (169-179)	18 Greece	18 Cyprus	153 (141-166)	-6·4 (-11 to -1·5)
171 (167-174)	19 Belgium	19 France	148 (135-163)	-3·1 (-9·1 to 3·3)
170 (167-174)	20 Portugal	20 Spain	146 (138-155)	-9·8 (-13 to -6·5)
163 (158-169)	21 Cyprus	21 Luxembourg	146 (130-164)	-8·8 (-15·6 to -1·4)
162 (159-165)	22 Spain	22 Greece	145 (135-156)	-16·8 (-20·4 to -12·8)
160 (155-165)	23 Ireland	23 Ireland	138 (123-154)	-14 (-20·6 to -6·9)
160 (154-166)	24 Luxembourg	24 Slovenia	136 (119-157)	-21·7 (-28·6 to -13·8)
159 (155-162)	25 United Kingdom	25 Malta	133 (116-152)	-13·2 (-20·6 to -4·7)
153 (149-157)	26 France	26 United Kingdom	127 (120-136)	-19·8 (-22·6 to -16·6)
153 (146-160)	27 Malta	27 Portugal	103 (95-112)	-39·4 (-43·1 to -35·3)
109 (105-113)	28 Italy	28 Italy	73 (66-82)	-32·9 (-37·5 to -27·9)

Figure VIII: Age- and sex-adjusted prevalence rate of stroke (per 10,000) in 2017 and 2047.

* adjusted to the 2013 European Standard Population.

2017 Age-adjusted rate* (95% CI)	2017 Country	2047 Country	2047 Age-adjusted rate* (95% CI)	2047 vs 2017 % Change (95% CI)
321 (307-336)	1 Bulgaria	1 Bulgaria	267 (225-318)	-16·8 (-26·7 to -5·2)
290 (273-307)	2 Romania	2 Romania	267 (199-362)	-7·8 (-27·2 to 17·9)
233 (214-255)	3 Latvia	3 Lithuania	181 (148-219)	-2·5 (-14·7 to 10·2)
186 (173-199)	4 Lithuania	4 Latvia	166 (118-241)	-28·8 (-44·6 to -5·4)
164 (154-175)	5 Croatia	5 Croatia	88 (74-106)	-46·4 (-52·1 to -39·7)
135 (128-142)	6 Hungary	6 Slovakia	61 (43-90)	-54·6 (-66·3 to -35·9)
134 (128-141)	7 Slovakia	7 Hungary	53 (45-63)	-60·5 (-64·8 to -55·3)
124 (116-134)	8 Poland	8 Greece	53 (43-68)	-54·5 (-60·5 to -46·2)
117 (108-126)	9 Greece	9 Malta	46 (38-55)	-48·3 (-53·9 to -41·7)
113 (108-119)	10 Portugal	10 Poland	42 (18-146)	-65·9 (-84·5 to 9·2)
112 (104-121)	11 Czech Republic	11 Cyprus	42 (35-51)	-44·5 (-50·3 to -37·6)
89 (84-94)	12 Malta	12 Sweden	40 (35-45)	-46·5 (-50·5 to -41·9)
86 (82-91)	13 Slovenia	13 Slovenia	38 (31-47)	-56·2 (-62·4 to -48·5)
78 (69-88)	14 Estonia	14 Denmark	36 (31-42)	-51·1 (-55·9 to -45·7)
76 (71-81)	15 Cyprus	15 Italy	34 (30-38)	-53·7 (-57·3 to -49·7)
75 (72-78)	16 Finland	16 Finland	33 (29-37)	-56 (-59·3 to -52·2)
75 (72-78)	17 Sweden	17 Portugal	33 (26-40)	-71·3 (-75·5 to -66·1)
74 (71-78)	18 Denmark	18 Czech Republic	31 (24-40)	-72·3 (-76·7 to -66·8)
73 (70-76)	19 Italy	19 Ireland	30 (23-38)	-55·2 (-62·9 to -45·5)
69 (65-72)	20 Luxembourg	20 Netherlands	30 (25-35)	-54·2 (-58·7 to -49·2)
68 (65-71)	21 United Kingdom	21 United Kingdom	30 (26-34)	-56·4 (-59·7 to -52·4)
67 (64-70)	22 Belgium	22 Belgium	29 (26-33)	-56·6 (-59·6 to -53·2)
67 (63-70)	23 Ireland	23 Luxembourg	22 (18-26)	-68·2 (-72 to -63·6)
65 (61-69)	24 Netherlands	24 Germany	20 (16-27)	-67·6 (-72·2 to -59·4)
62 (58-66)	25 Germany	25 France	19 (16-23)	-62·3 (-67·5 to -55·7)
57 (55-58)	26 Spain	26 Spain	18 (17-20)	-67·8 (-69·8 to -65·6)
50 (48-53)	27 France	27 Estonia	11 (7-17)	-85·9 (-89·6 to -80·8)
50 (47-53)	28 Austria	28 Austria	10 (9-12)	-79·5 (-81·2 to -77·6)

Figure IX: Age- and sex-adjusted mortality rate of stroke (per 100,000) in 2017 and 2047.

* adjusted to the 2013 European Standard Population.

2017		2047		2047 vs 2017
Age-adjusted rate* (95% CI)	Country	Country	Age-adjusted rate* (95% CI)	% Change (95% CI)
502 (485-520)	1 Bulgaria	1 Romania	429 (323-575)	-7 (-25·9 to 17·8)
461 (436-488)	2 Romania	2 Bulgaria	346 (304-398)	-31 (-37·3 to -23·5)
382 (354-412)	3 Latvia	3 Lithuania	293 (243-367)	-8·7 (-20·1 to 8·4)
320 (304-339)	4 Lithuania	4 Latvia	199 (158-253)	-47·9 (-55·3 to -38·6)
264 (249-280)	5 Croatia	5 Croatia	129 (111-152)	-51 (-55·6 to -45·9)
241 (232-250)	6 Slovakia	6 Slovakia	114 (88-150)	-52·8 (-62·1 to -40·2)
238 (229-248)	7 Hungary	7 Hungary	91 (81-103)	-61·7 (-64·7 to -58·4)
218 (206-231)	8 Poland	8 Poland	74 (40-159)	-66·3 (-80·6 to -31·1)
178 (169-187)	9 Czech Republic	9 Greece	70 (61-82)	-56·2 (-60·1 to -51·5)
160 (145-178)	10 Estonia	10 Malta	56 (48-66)	-53·2 (-57·8 to -48)
160 (152-169)	11 Greece	11 Sweden	55 (50-60)	-49·4 (-52·3 to -46·4)
153 (146-160)	12 Portugal	12 Cyprus	53 (46-62)	-52·5 (-56·8 to -47·7)
133 (127-140)	13 Slovenia	13 Finland	52 (48-57)	-56·9 (-59 to -54·6)
121 (118-125)	14 Finland	14 Denmark	50 (44-56)	-54·6 (-58·2 to -50·7)
120 (114-127)	15 Malta	15 Czech Republic	49 (42-57)	-72·5 (-75·2 to -69·2)
112 (106-119)	16 Cyprus	16 Netherlands	47 (41-54)	-52·8 (-56·8 to -48·3)
110 (106-114)	17 Denmark	17 Belgium	46 (43-50)	-55·7 (-57·9 to -53·3)
109 (106-112)	18 Sweden	18 Slovenia	46 (39-54)	-65·5 (-68·9 to -61·5)
104 (101-108)	19 Belgium	19 United Kingdom	40 (36-44)	-60·1 (-62·3 to -57·6)
101 (96-106)	20 Luxembourg	20 Portugal	37 (31-44)	-75·8 (-78·8 to -72·3)
100 (95-105)	21 Germany	21 Germany	37 (32-44)	-63 (-66·7 to -58·1)
100 (95-105)	22 Netherlands	22 Italy	35 (31-39)	-62 (-64·5 to -59·3)
99 (96-103)	23 United Kingdom	23 Ireland	34 (28-43)	-63·4 (-68·8 to -56·7)
94 (89-99)	24 Ireland	24 France	34 (30-39)	-58·6 (-62·4 to -54·2)
92 (88-95)	25 Italy	25 Luxembourg	30 (26-35)	-70·1 (-73 to -66·7)
87 (85-90)	26 Spain	26 Spain	29 (27-32)	-66·3 (-67·9 to -64·5)
86 (81-91)	27 Austria	27 Estonia	26 (18-38)	-83·5 (-87·3 to -78·5)
82 (79-85)	28 France	28 Austria	23 (20-26)	-73·2 (-74·9 to -71·2)

Figure X: Age- and sex-adjusted rate of DALYs lost because of stroke (per 10,000) in 2017 and 2047.

* adjusted to the 2013 European Standard Population.
